# A Configurable Architecture for Two Degree-of-Freedom Variable Stiffness Actuators to Match the Compliant Behavior of Human Joints

**DOI:** 10.3389/frobt.2021.614145

**Published:** 2021-03-12

**Authors:** Simon Lemerle, Manuel G. Catalano, Antonio Bicchi, Giorgio Grioli

**Affiliations:** ^1^ Centro “E. Piaggio” and Dipartimento di Ingegneria dell’Informazione, University of Pisa, Pisa, Italy; ^2^ Soft Robotics for Human Cooperation and Rehabilitation, Fondazione Istituto Italiano di Tecnologia, Genoa, Italy

**Keywords:** artificial joints, articulated soft robotics, variable stiffness, humanoids, prostheses

## Abstract

Living beings modulate the impedance of their joints to interact proficiently, robustly, and safely with the environment. These observations inspired the design of soft articulated robots with the development of Variable Impedance and Variable Stiffness Actuators. However, designing them remains a challenging task due to their mechanical complexity, encumbrance, and weight, but also due to the different specifications that the wide range of applications requires. For instance, as prostheses or parts of humanoid systems, there is currently a need for multi-degree-of-freedom joints that have abilities similar to those of human articulations. Toward this goal, we propose a new compact and configurable design for a two-degree-of-freedom variable stiffness joint that can match the passive behavior of a human wrist and ankle. Using only three motors, this joint can control its equilibrium orientation around two perpendicular axes and its overall stiffness as a one-dimensional parameter, like the co-contraction of human muscles. The kinematic architecture builds upon a state-of-the-art rigid parallel mechanism with the addition of nonlinear elastic elements to allow the control of the stiffness. The mechanical parameters of the proposed system can be optimized to match desired passive compliant behaviors and to fit various applications (e.g., prosthetic wrists or ankles, artificial wrists, etc.). After describing the joint structure, we detail the kinetostatic analysis to derive the compliant behavior as a function of the design parameters and to prove the variable stiffness ability of the system. Besides, we provide sets of design parameters to match the passive compliance of either a human wrist or ankle. Moreover, to show the versatility of the proposed joint architecture and as guidelines for the future designer, we describe the influence of the main design parameters on the system stiffness characteristic and show the potential of the design for more complex applications.

## 1 Introduction

In the last years, articulated soft robots inspired by the musculoskeletal system of vertebrate animals received increased attention from researchers, since they represent promising solutions to enhance the interactions of robots with unknown and dynamic environments, i.e., the real world (Albu-Schaeffer et al., 2008). In line with this, Variable Impedance Actuators and the subgroup of Variable Stiffness Actuators (VSAs) were widely investigated recently (Vanderborght et al., 2013).

Variable Stiffness Actuators usually rely on two main approaches to modulate the stiffness. In the first one, the stiffness is controlled using software together with a mechanism with fixed impedance properties. In the other one, the stiffness is modulated through a mechanical reconfiguration of the system (Vanderborght et al., 2013). Some literature refers to the first approach as *active VSA* and the second one as *passive VSA* (Vanderborght et al., 2013). However, to avoid confusion possibly induced by the notion of passivity, we will refer to the first approach as *software-controlled VSA* and to the second one as *physically compliant VSA*. Software-controlled VSAs allow the design of lightweight devices that can in theory simulate any desired stiffness behavior. However, this apparent stiffness emulated by the system relies on an accurate sensing strategy and control computations, and it has been shown that even if the impacts are detected timely, the motors could not be able to react fast enough by solely an impedance control and that the system should therefore be considered as stiff during the impacts (Haddadin et al., 2007). To address these limitations, physically compliant VSAs have been developed with an inherent compliance. They can present several advantages such as shock absorptions, better performances in cyclic or explosive tasks (Albu-Schaeffer et al., 2008; Wolf et al., 2016), and a possible embodiment of specific behavior to improve the control strategies (Visser et al., 2011).

As VSAs are inspired by musculoskeletal systems, their applications as prostheses or part of humanoid devices would seem straightforward. Even though there are some advantages in using VSAs compared to stiff actuators, finding concrete use-cases of VSAs is an on-going research topic and requires attention (Sensinger and ff. Weir, 2008; Blank et al., 2014; Stillfried et al., 2018).

To test hypotheses in this objective, we need multi-degree-of-freedom (DoF) joints that show abilities similar to those of a human joint, such as the same functional range of motion, a variable stiffness mechanism, the overall shape and mass, and so on. Integrating an inherent compliance inside these systems enables us to test both the variable stiffness ability but also the capabilities due to the compliance, such as the possibility of exploiting the natural dynamic of the system (e.g., the resonance frequency) (Stillfried et al., 2018). Besides, artificial wrists can benefit from a compliant architecture to enhance their manipulation abilities in tight or cluttered spaces (Negrello et al., 2019). Moreover, the availability of various levels of stiffness can enhance the performances in activities of daily living in prosthetic applications (Kanitz et al., 2018). However, the mechanical complexity of such mechanisms increases and constitutes a challenge.

Up to now, most of the proposed physically compliant VSAs have only 1 DoF in position (Vanderborght et al., 2009; Vanderborght et al., 2013; Petit et al., 2015; Lemerle et al., 2019). The classical approach to get multi DoF physically compliant VSAs is to put in series several 1-DoF VSAs, such as the solutions proposed in the DLR Hand Arm System (Grebenstein et al., 2011; Romano et al., 2014; Savin et al., 2019). This approach uses the design of serial manipulators (SMs) that are easier to model and control compared to the parallel manipulators (PMs). The latter, on the other hand, allow more compact designs and have better performances in terms of output torques than SMs for the same motor sizes. Nevertheless, the performances of PMs are very sensitive to their geometric parameters (Siciliano and Khatib, 2007). Alternative works explored the independent setup approach, consisting mainly of a serial arrangement of position motor units with several DoF in position (using either SMs or PMs) and an additional motor unit dedicated solely to the control of the stiffness (Weckx et al., 2014; Barrett et al., 2017; Tan et al., 2017; Rodriguez-Cianca et al., 2019). Finally, other works draw inspiration from the antagonistic arrangements of the musculoskeletal system (Koganezawa, 2017; Stoeffler et al., 2018; Malosio et al., 2019). In the last category, several antagonistic motors are used jointly to control the stiffness and the position of the joint. Theses architectures are solely based on PMs. For more details on the classification and principle of work of VSAs, the reader is invited to refer to (Vanderborght et al., 2013; Wolf et al., 2016).

One limitation of physically compliant VSAs is that one mechanical implementation corresponds to one embedded passive behavior. Although there are devices that are human-like in terms of shapes and dimensions, and are able to perform activities of daily living (ADL) (Grebenstein et al., 2011; Weckx et al., 2014; Koganezawa, 2017; Stoeffler et al., 2018; Rodriguez-Cianca et al., 2019), there is, for now, no multi-DoF VSA embedding a compliant behavior that matches human joints. It is worth mentioning that there are alternatives to design compact 2 DoF joints with inherent compliance (not necessarily with variable stiffness in the following examples). A first concept is to design an underactuated mechanism (Casini et al., 2017). Its main drawback could be its limited dexterity to do all the desired tasks. Another approach is to have a switchable stiffness to get lightweight and compact devices (Montagnani et al., 2013). This type of solution could be suitable when lightweight systems are needed. However, they do not allow for a continuous and smooth control of the stiffness. Therefore, they lose some advantages of such abilities, e.g., during explosive or cyclic tasks, when a VSA performs better than a fixed compliant mechanism (Garabini et al., 2011). An additional idea is to use tendon driven mechanisms with compliant pneumatic actuation (Toedtheide and Haddadin, 2018). Then, this system could be lightweight and somehow compact if the remote actuation units are excluded. Yet, this is not fully satisfying in terms of compactness if we consider them, especially when they are pneumatic actuation units. Therefore, all of these approaches do not fully satisfy the criteria of compactness and variable stiffness ability as well as doing various types of tasks. We address this limitation in the following work.

In general, the qualitative representation of the mechanical impedance of human joints is made using stiffness (or compliance) ellipsoids (Mussa-Ivaldi et al., 1985). To modulate the stiffness ellipsoids of the end effector during tasks, it appears that the limb geometry and posture have a large impact on its shape and orientation, whereas the voluntary co-contraction of the participating muscles affects mainly the size of the ellipsoids (Milner, 2002; Perreault et al., 2002). To quantify the mechanical impedance properties of multi-DoF human joints, the traditional approach consists in applying position or force perturbations to the joint of interest and measuring the displacement force or position responses (Mussa-Ivaldi et al., 1985). Yet, this process is complex and long. Investigations are made to simplify and speed up the procedure (Masia et al., 2012; Masia and Squeri, 2015). The measurements usually concern the endpoint impedance (Mussa-Ivaldi et al., 1985; Masia et al., 2012). Yet, there is mechanical impedance data of specific human joints, such as the wrist (Formica et al., 2012; Pando et al., 2014) or the ankle (Kearney et al., 1997; Lee et al., 2014). There is, for now, no data on the mechanical impedance of more complex joints such as the shoulder or hip. Yet, efforts are made toward this direction with the design of low inertia shoulder exoskeleton to measure neuromuscular properties (Hunt and Lee, 2019).

The observations on how human beings modulate the stiffness ellipsoids were successfully exploited to implement a teleimpedance controller to generate human-like motions and desired task space impedance (Ajoudani et al., 2018). Similarly, these observations could be used to reduce the mechanical complexity of human-like VSAs. Indeed, if the human-like compliant behavior is directly implemented in the mechanism, only one additional actuator is ideally required to modulate the overall size of the stiffness ellipsoid, in analogy to the voluntary co-contraction of human muscles. As a matter of fact, from the study of tendon mechanisms (e.g., the Salisbury Hand), we know that to control N DoF in position only N+1 actuators are required. There is then an internal tension that can be used to modulate the stiffness. This was shown for a grasping hand in Mason and Salisbury (1985).

**FIGURE 1:**
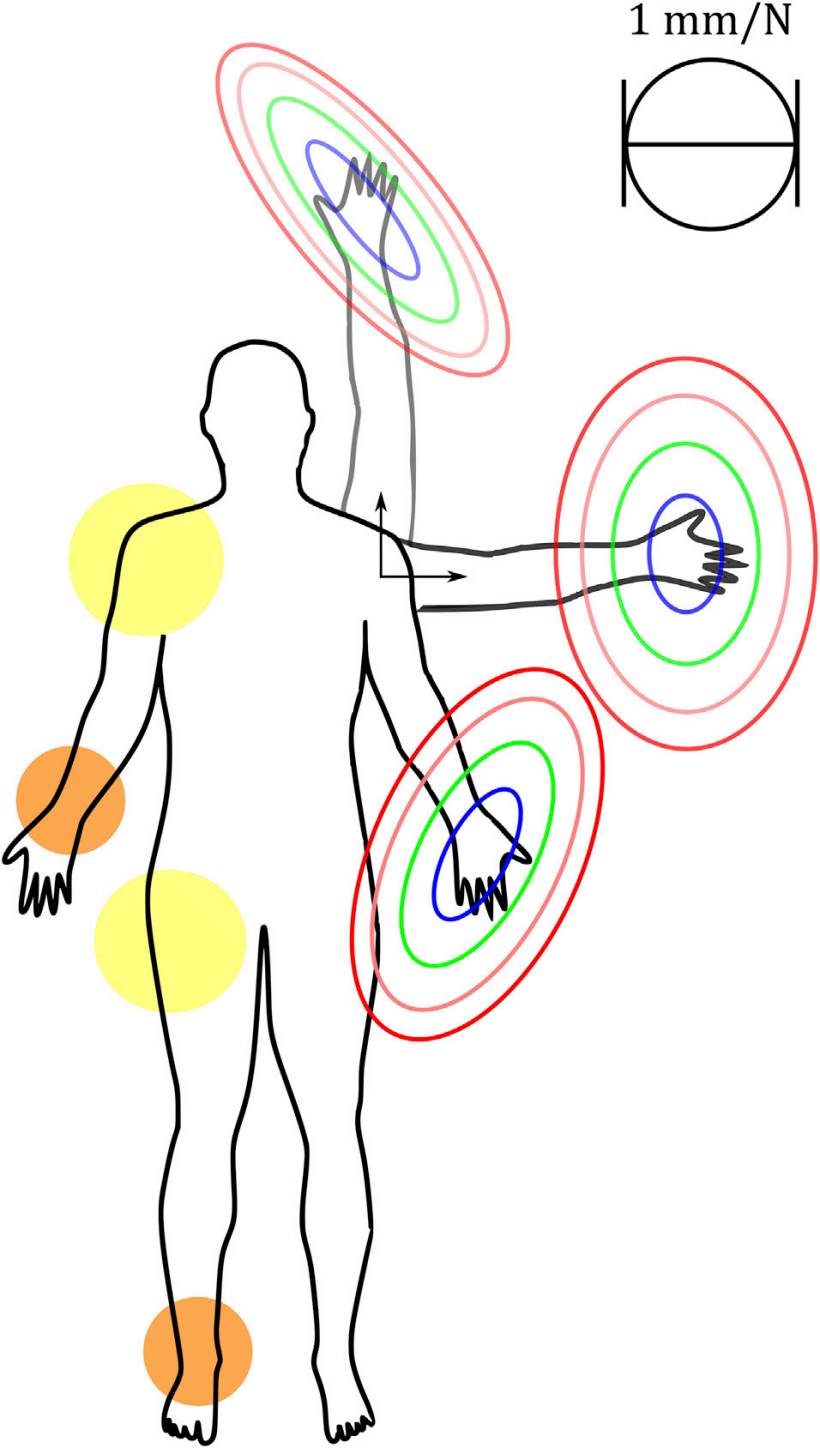
The proposed 2-DOF variable stiffness joint allows shaping the stiffness (or compliance) ellipsoid at different postures in the design phase, while the volume can be varied in real-time to make the overall behavior stiffer or softer. The kinematics of the 2-DOF VS joint can be used for the design of wrists and ankles of human-like bionic systems (in orange). In principle, this joint can also be used as part of more complex designs for a shoulder or hip human-like joint (in yellow).

In this work, we propose the concept and modeling of a configurable architecture of a physically compliant 2 DoF VSA that can match the passive behavior of either a human wrist or a human ankle. This joint can control its orientations around 2 perpendicular axes and its overall stiffness as a one-dimensional parameter similar to the co-contraction of human muscles. It is based on a state-of-the-art parallel manipulator that we modified to implement an inherent compliance with a compact architecture. Hence, the variable stiffness (VS) ability of the system relies on the antagonistic arrangement of nonlinear elastic actuators (Vanderborght et al., 2013; Wolf et al., 2016). The compact architecture of the system is based on the PM architecture with the minimum number of required motors to get a VSA for 2 DoF in position (i.e., 3). Thanks to its kinematics, the proposed system could be used as a wrist or ankle joint. It can also be part of more complex joints such as the hip or shoulder joint. Indeed, these joints are represented with 3 DoF as ball-and-socket joints and one of their three rotations is around a longitudinal axis of the body segment attached to the joint (Wilhelms and Gelder, 2001). Therefore, we can design them with a hybrid architecture of a PM with 2 DoF in series with a 1 DoF rotator, like for instance the humeral rotator proposed in Mazzotti et al., (2015), which can be integrated with a 2 DoF shoulder joint presented in Troncossi et al., (2009). A similar idea could be proposed for a hip joint with a femoral rotator. Therefore, our proposed system could also be used as part of these more complex joints. Figure 1 shows the general principles of the system and some of its potential applications.

Besides the general description of the proposed system in Section 2.1, the main contributions of this paper are: (i) to prove the human-like VS ability of the system with a compact architecture (Section 4.1), (ii) to study the effect of the main design parameters of the system on its passive compliance to provide general guidelines (Section 4.2), and (iii) to provide sets of design parameters to match the passive compliance of a human wrist or ankle based on an optimization process (Section 4.3). Other contributions of this paper include improvements on the kinematic analysis of the rigid structure (used as a basis of our mechanical architecture) done in Sofka et al., (2006a) and the kinetostatic analysis of our proposed compliant structure (Section 3).

The paper is organized as in the following: Section 2 details the design concept of the proposed system and the methodology of its study. It includes, in particular, a description of its general architecture and its main design parameters. Section 3 provides the model and the kinetostatic analysis of the system. Section 4 gives the main results of the current study that are then discussed in Section 5. Finally, Section 6 summarizes the main contributions and concludes the article.

## 2 Design Concept and Methodology

### 2.1 General Architecture of the System

**FIGURE 2:**
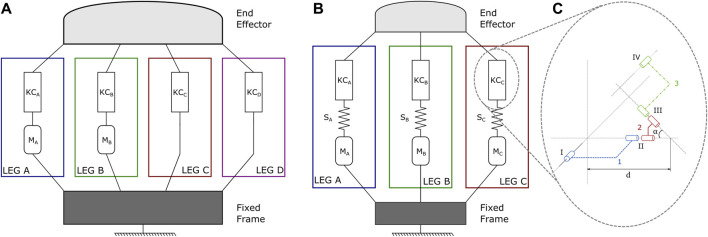
**(A)** is the classic architecture of the Omni-Wrist III (Rosheim and Sauter, 2002) is composed of 4 legs linking a fixed frame and a moving platform. Each actuated leg is composed of the serial arrangement of one motor $\left(M_{\text{*}}\right)$ and one kinematic chain $\left(KC_{\text{*}}\right)$. Each non-actuated leg is composed of one kinematic chain (KC_*_) **(B)** Represents the architecture of the proposed system. We use a configuration with 3 legs only. In each leg, we add elastic elements $\left(S_{\text{*}}\right)$ between the motor $\left(M_{\text{*}}\right)$ and the kinematic chain $\left(KC_{\text{*}}\right)$. **(C)** shows the details of one kinematic chain (each KCs are identical). It is composed of 3 linkages (1, 2 and 3) and 4 noncoplanar revolute joints (I, II, III, IV). The joint IV is linked to the end effector and the joint I is linked either to the output of the motor in the Omni-Wrist III or to the output of the elastic element in our proposed architecture.

Based on the state of the art of compact 2 DoF in orientation joints based on PMs (refer to (Bajaj et al., 2019) for a quite extended list), we selected the kinematic architecture of the Omni-Wrist III, designed by Rosheim and Sauter (2002) and studied by Sofka et al., (2006a) and Sofka et al., (2006b). This design has several advantages such as 180° hemispherical singularity free movement (Rosheim and Sauter, 2002). In theory, only two legs are needed to actuate the system but the main version has 4 legs. Its general architecture is shown in Figure 2A. In our design, we propose to use a 3–legs configuration with nonlinear elastic elements added in the serial arrangement of each leg. Figure 2B presents its general architecture. The system is a PM composed of a fixed frame (in dark gray on the scheme) and a moving platform (in light gray), i.e., the end effector, linked together with three independent and identical kinematic serial chains, called respectively leg A (in blue), leg B (in green) and leg C (in red). Each leg is composed of the serial arrangement of one motor $\left(M_{*}\right)$, one nonlinear elastic element $\left(S_{*}\right)$, and one kinematic chain $\left(KC_{*}\right)$. The kinematic chains, based on the geometry proposed in Rosheim and Sauter (2002), are composed of 3 linkages each connected together and to the end effector by a succession of 4 noncoplanar revolute joints. Based on the modeling of Sofka et al., these KCs are parametrized by *α*, which is the angular deviation of the link 2 (in red), and by a characteristic length *d* (Sofka et al., 2006a). Figure 2C shows the main structure of one KC with its parameters.

### 2.2 Variable Stiffness Ability

As it is common in VSA with antagonistic architecture, to get the variable stiffness ability, the stiffness characteristic of the elastic element has to be nonlinear. There are many ways to design nonlinear springs, to cite a few, the physical implementation can rely on tendons and linear springs (Catalano et al., 2011), on a combination of belts and linear springs (Lemerle et al., 2019), on a combination of torsional spring with guide shafts with varying radius (Koganezawa et al., 2005; Koganezawa, 2017), or on hydraulic or pneumatic solutions (Stoeffler et al., 2018).

Nevertheless, nonlinear stiffness alone is not sufficient to grant the variable stiffness control of the system, which is equivalent to prove that we can control independently the equilibrium orientations of the system and its overall stiffness along one dimension. To prove this analytically, the kinetostatic analysis of the system is first presented in Section 3. Then, the final arguments to show the VS control of the proposed system are given in Section 4.1.

In addition, we would like to show the effects of the modulation of the stiffness on the overall compliance of the system. To represent them graphically, we need to derive a model of the system (done in Section 3) and to model the elastic elements. In this study, we make the deliberate choice of adopting a hyperbolic sine spring, whose mechanical characteristics have the form

$$f_{S_{\text{*}}}=2K_{\text{*}}\sinh\left(\delta_{\text{*}}/\delta_{0_{\text{*}}}\right),$$ (1)

where $f_{S_{\text{*}}}$ stands for the force exerting by the spring, $K_{\text{*}}$ for the spring constant, $\delta_{\text{*}}$ the deflection of the spring, and $\delta_{0_{\text{*}}}$ a characteristic deflection of the spring. This choice is motivated by the fact that springs with a similar characteristic have been successfully used in other actuators (Catalano et al., 2011; Lemerle et al., 2019), and recent works suggested that this type of elastic behavior replicate several attributes of an antagonistic pair of muscles driving a joint (Garabini et al., 2017). This restricts the conclusions drawn in the next sections to be exact only when dealing with this specific kind of springs. Nevertheless, it is expected to observe similar trends in all springs with similar stress-stiffening characteristics.

For the sake of clarity, the detailed graphical representation methodology is described after the complete analysis of the system in Section 3.8. The effects of the modulation of the stiffness are shown in Section 4.1.

### 2.3 General Design Guidelines

**FIGURE 3:**
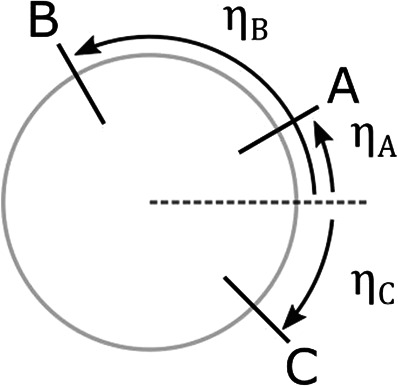
Scheme of the leg configuration with their angular positions.

As previously described, there are various ways to implement the nonlinear elastic elements. In a first approach, we leave this choice to the designer to focus on the design of the KCs and their arrangement. Therefore, as general design guidelines, we study the effects of the purely geometric parameters (simply referred to as geometric parameters) on the compliance of the proposed system. To do this study, the compliance of the system should be modeled as a function of these geometric parameters. This is done in Section 3. The first step in the modeling is to define the geometric parameters. They correspond to the parameters of the KCs *α* and *d* (Sofka et al., 2006a), and the angular arrangement of the first joint of each leg around the basis, namely $\eta_{A}$, $\eta_{B}$, $\eta_{C}$ (refer to the parametrization of the system in Section 3.1 for the exact definitions of these parameters). The angular positions of each leg are defined according to the scheme shown in Figure 3.

In the case of a fixed distribution of the legs around the basis, *α* and *d* are not enough to modulate the compliant behavior of the system (refer to Section 4.2 for more details). That is why we need to extend the modeling done in Sofka et al. (2006a) by adding the angular positions of the legs.

Moreover, to study separately the effects on the compliance due to the kinematic architecture from the effects due to the internal variation of the compliance of the elastic elements, the compliance characteristic of each leg is modeled as a constant. Concretely, to study the impact of the angular position of the legs, two types of configuration are simulated. Firstly the leg A is considered as fixed with $\eta_{A}=0$ and the couple ($\eta_{B}$, $\eta_{C}$) is varying symmetrically, with $\eta_{B}$ in {π/4, π/3, π/2, 2π/3, 3π/4} and $\eta_{C}=-\eta_{B}$. Secondly, the legs A and C are fixed, respectively equal to π/4 and −π/4 and $\eta_{B}$ is varying in the set {−3π/4, −2π/3, −π/2, −π/3, π/3, π/2, 2π/3, 3π/4, *π*}. To study the impact of the angular deviation of the middle link of each leg *α*, this angle is varying in the set {π/6, π/4, π/3, 5π/12}. Finally, to study the impact of the characteristic length *d* of each leg, this length is varying in the set {25 mm, 50 mm, 75 mm, and 100 mm}. All the simulations and computations are performed using MATLAB. The results are shown in Section 4.2.

### 2.4 Design Parameters to Match Compliance of Human Wrist and Ankle

One of the main goals of this study is to show that we can implement passive compliant behaviors of human joints. In this work, we focus on two different human joints: the wrist and the ankle. To find the appropriate sets of design parameters to match their passive compliant behavior, we derived an optimization process. In the first step in Section 2.4.1, we need to define the behaviors we would like to embed based on the mechanical impedance data of human joints. Then, in Section 2.4.2 we define the design parameters of the proposed architecture. Finally, to derive the cost function of the minimization algorithm, we need to characterize the compliance of the system as a function of the design parameters. This is done in Section 3 and the cost function is defined in Eq. 58. Results of the optimization process are shown in Section 4.3.

#### 2.4.1 Parameters of Human Wrist and Human Ankle

Based on the state-of-the-art, we selected the biomechanical data of a human wrist presented in Pando et al. (2014) and of a human ankle presented in Lee et al. (2014).

In Pando et al. (2014), Pando et al. studied, among others, the passive wrist stiffness in the two dimensions that are the flexion/extension and the radial/ulnar deviation. They extracted from their data the stiffness ellipses around the neutral position of the wrist in case of no voluntary co-contraction of the subjects to evaluate thus the passive stiffness of the articulation. They found that the human wrist stiffness is anisotropic and the direction of the greater magnitude of the average stiffness ellipse is around 12.1° from the pure radial deviation in the counter-clockwise direction toward a pure flexion. The ratio between the magnitude of the two axes of the stiffness ellipse is about 2.69 and using the area of the ellipse [5.61 (Nm/rad)^2^], we can extract the lengths of the two semi-axes of the ellipse, which are then 2.19 and 0.81 Nm/rad.

In Lee et al. (2014), Lee et al. studied the mechanical impedance of a human ankle with relaxed muscles, i.e., in passive conditions, both in seated and standing positions. They found that for low-frequency motion the stiffness dominates the mechanical impedance of the ankle, which is our application case as we consider the static stiffness in our design. Its characteristics in standing positions are: a tilt angle of 10°, a magnitude in the major principal axis direction of 23.86 Nm/rad, and a magnitude in the orthogonal direction of 8.14 Nm/rad (the ratio is 2.99).

Considering these data, we can derive the average Cartesian compliance ellipse and the associated compliance matrix for both a human wrist and ankle, using similar parametrization as the one proposed in Section 3.7.

#### 2.4.2 Design Parameters Definition

In a second step, we should define all the design parameters of the proposed architecture. They can be divided into geometric parameters (previously described), elastic parameters, and internal modulation parameters. As a reminder, there are 5 geometric parameters: *α*, *d*, $\eta_{A}$, $\eta_{B}$ and $\eta_{C}$.

Each nonlinear spring can be characterized by nonlinear functions. Their explicit formulations depend on mechanical implementation. They can have various forms such as polynomial functions of degree higher than 2 (e.g., quadratic springs), trigonometric functions, exponential or hyperbolic functions to name but a few (English and Russell, 1999; Koganezawa et al., 2005; Vanderborght et al., 2009; Catalano et al., 2011; Petit et al., 2015; Lemerle et al., 2019). Therefore the number of their associated parameters depends on the complexity of the model. These parameters are referred to as elastic parameters. Noting $n_{elastic}$ the number of parameters for one nonlinear spring, there are in total $3\times n_{elastic}$ elastic parameters in the proposed system. They can be highly variable depending on the mechanical implementation. Moreover, it is not easy to derive realistic values of them to fix the bound constraints for the minimization problem. Therefore, it is better if the solution provided by the algorithm is robust or independent with the values of these parameters.

The internal modulation parameter, called *λ*, stands for the one-dimensional quantity we can use to control the stiffness in the proposed system. Unlike the previous parameters, *λ* is not fixed to one mechanical implementation and can therefore be different if we try to match several compliant behaviors. Therefore, there is one additional parameter for each desired behavior.

Therefore, the number of design parameters, noted $n_{param}$, to match the specified targeted behaviors is defined as

$$n_{param}=5+3\times n_{elastic}+n_{targets},$$ (2)

where $n_{elastic}$ stands for the elastic parameters of one leg and $n_{targets}$ the number of compliant targeted behaviors.

For this study, we model the nonlinear elastic elements as hyperbolic sine springs for similar reasons as previously described in Section 2.2. Their mechanical characteristics have the form defined in Eq. 1. Therefore, there are 2 elastic parameters for each leg: (i) the spring constant $K_{\text{*}}$ and (ii) the free length $\delta_{0\text{*}}$. All the design parameters are summarized in Table 1.

**TABLE 1:** List of design parameters.

Symbol	Description
*α*	Characteristic angular deviation of each leg
*d*	Characteristic length of each leg
$\eta_{A}$, $\eta_{B}$ and $\eta_{C}$	Angular orientations of each leg
$K_{\text{*}}$ and $\delta_{0_{\text{*}}}$	Elastic parameters
$\lambda \in {\mathbb{R}}^{n_{targets}}$	Internal modulation parameter
	$n_{targets}$ stands for the number of desired compliant behavior.

## 3 Model and Analysis

In this section, we describe the kinetostatic analysis of the proposed system with two goals in mind: (i) to derive the compliance matrix as a function of the design parameters and (ii) to show the variable stiffness ability of the system.

Firstly the kinematic analysis done by Sofka et al. (2006a) needs to be extended to include the angular arrangement of the legs. Then, we describe the inverse and differential kinematics of the system in a closed form, showing that it is a 2 DoF joint. We provide an explicit and complete formulation of the general mapping of the generalized pose of the end effector as a function of a minimal parametrization. This result is then used to analyze the static equilibrium of the system and describe its static stiffness and Cartesian compliance, required for the optimization algorithm. A list of the main symbols used in the analysis can be found in Table 2.

**TABLE 2:** List of main symbols.

Symbol	Description
$C_{0}=\left[{ℬ}_{0},O_{0}\right]$	Reference coordinate frame attached to the fixed base
$C_{ee}=\left[{ℬ}_{ee},O_{ee}\right]$	Reference frame attached to the end effector
$O_{0}$, $O_{ee}$	Origins of the frames $C_{0}$ and $C_{ee}$
${ℬ}_{0}=\left[X_{0},Y_{0},Z_{0}\right]$	Orthonormal basis of the frame $C_{0}$
${ℬ}_{ee}=\left[X_{ee},Y_{ee},Z_{ee}\right]$	Orthonormal basis of the frame $C_{ee}$
x	Generalized pose of the end effector
${\left(\begin{array}{ccc} \alpha_{x} & \alpha_{y} & \alpha_{z} \end{array}\right)}^{T}$	Angular orientations of the end effector
${\left(\begin{array}{ccc} x_{ee} & y_{ee} & z_{ee} \end{array}\right)}^{T}$	Cartesian position of $O_{ee}$
Tji	Transformation matrix from $C_{i}$ to $C_{j}$
Tlji	Transformation matrix from $C_{i}$ to $C_{j}$ through leg *l*
$\forall i\in \left\{1,2,3,4\right\},\;q_{i}$	Angular position of $i^{\mathrm{th}}$ joint for a generic leg
*α*	Angular deviation of the middle link of each leg
*d*	Characteristic length of each leg
*η*	Generic angular orientation of one leg
$\eta_{l}$	Specific angular orientation of leg *l*
${\left(g_{i}\right)}_{i\in \left\{1,\ldots ,7\right\}}$ and ${\left(h_{i}\right)}_{i\in \left\{1,2,3\right\}}$	families of functions defined respectively in Eqs. (11) and (12)
$\forall i\in \left\{A1,\ldots ,C4\right\},\;q_{i}\text{ and }\tau_{i}$	Specific angular position and torque of joint *i*
$u={\left(\begin{array}{cc} \alpha_{y} & \alpha_{z} \end{array}\right)}^{T}$	Minimal parametrization of the system
*T*	General mapping of the system
*L*	Distance between $O_{0}$ and $O_{ee}$
$q_{act}={\left(\begin{array}{ccc} q_{A1} & q_{B1} & q_{C1} \end{array}\right)}^{T}$	Positions of the actuated joints
$\tau_{act}$	Torques of the actuated joints
$f_{IK}:\;{\mathbb{R}}^{2}\to {\mathbb{R}}^{3}$	Inverse kinematics function
$A_{u}\in {\mathbb{R}}^{3\times 2}$	Jacobian matrix of $f_{IK}$ (dependent u)
$\tau_{u}\in {\mathbb{R}}^{2}$	Torque generated by the system
$\forall \text{*}\in \left\{A,B,C\right\},\;f_{S_{\text{*}}}$	Torque function of the elastic element of respectively leg *A*, *B* and *C*
$\forall \text{*}\in \left\{A,B,C\right\},\;\delta_{\text{*}}$	Deflection of the elastic element of respectively leg *A*, *B* and *C*
$\forall \text{*}\in \left\{A,B,C\right\},\;\theta_{M_{\text{*}}}$	Position of motor $M_{\text{*}}$
$N_{0}$	Basis of the solution space of the equation $A_{u}^{T}X=0$
$\sigma_{ee}$	Static stiffness of the system
K	Diagonal matrix of the stiffness of each leg
$p\in {\mathbb{R}}^{3}$	Cartesian position of a point attached to the end effector
$J\in {\mathbb{R}}^{3\times 2}$	Jacobian matrix of *p* with respect to u
$c\in {\mathbb{R}}^{3\times 3}$	Cartesian compliance of *p*
$c_{ref}\in {\mathbb{R}}^{3\times 3}$	Desired Cartesian compliance matrix
$\forall k\in \left\{x,y,z\right\},\;c_{k}\text{ and }s_{k}$	$\text{cos}\left(\alpha_{k}\right)$ and $\text{sin}\left(\alpha_{k}\right)$
$\forall i\in \left\{1,2,3,4\right\},\;c_{i}\text{ and }s_{i}$	$\text{cos}\left(q_{i}\right)$ and $\text{sin}\left(q_{i}\right)$
$\forall i\in \left\{A1,\ldots ,C4\right\},\;c_{i}\text{ and }s_{i}$	$\text{cos}\left(q_{i}\right)$ and $\text{sin}\left(q_{i}\right)$
$c_{\alpha }\text{ and }s_{\alpha }$	cos(α) and sin(α)
$\forall \text{*}\in \left\{A,B,C\right\},\;c_{\text{*}}\text{ and }s_{\text{*}}$	$\text{cos}\left(\eta_{\text{*}}\right)$ and $\text{sin}\left(\eta_{\text{*}}\right)$

### 3.1 Parametrization of the System

Define the reference coordinate frame attached to the fixed base of the mechanism

$$C_{0}=\left(X_{0},Y_{0},Z_{0},O_{0}\right),$$

where $O_{0}$ marks its origin and $\left(X_{0},Y_{0},Z_{0}\right)$ form an orthonormal basis, noted also ${ℬ}_{0}$. Define also, with analogous notation, the reference frame attached to the end effector of the mechanism

$$C_{ee}=\left(X_{ee},Y_{ee},Z_{ee},O_{ee}\right).$$

A possible choice of parameters that easily parametrizes the generalized pose of the end effector is

$$x={\left(\begin{array}{cccccc} \alpha_{x} & \alpha_{y} & \alpha_{z} & x_{ee} & y_{ee} & z_{ee} \end{array}\right)}^{T}\;,$$ (3)

where the first three components stand for the Euler angles of the end effector, defining, therefore, its angular orientation about respectively the axis $X_{0}$–$Y_{0}$–**Z**_0_, namely (roll, yaw, pitch) and the last three components are the position of its center $O_{ee}$ in ${ℬ}_{0}$.

Denoting with Rot(z,θ) and Trans(z,d) the homogeneous matrices associated to the transformations of a simple rotation of θ around the axis $\vec{z}$, and of a simple translation of *d* along the axis $\vec{z}$, respectively, it is possible to write

Cee=CTee0,

where

Tee0=Trans(Z0,zee)Trans(Y0,yee)Trans(X0,xee)Rot(Z0,αz)Rot(Y0,αy)Rot(X0,αx), (4)

i.e.,

Tee0=(czcyczsxsy−szcxczsycx+szsxxeeszcyszsxsy+czcxszsycx−czsxyee−sysxcycxcyzee0001) , (5)

where, $c_{k}$ and $s_{k}$ stand respectively for $\text{cos}\left(\alpha_{k}\right)$ and $\text{sin}\left(\alpha_{k}\right)$, for *k* in {x,y,z}.

**FIGURE 4:**
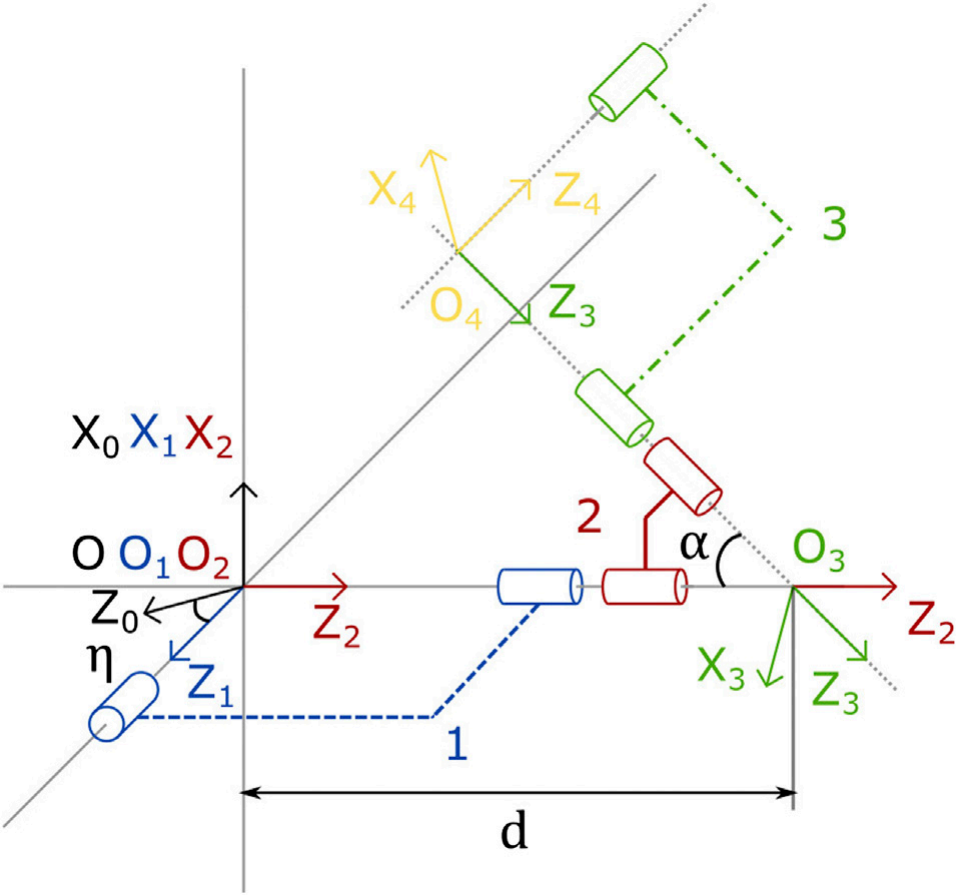
Kinematic diagram of a generic leg. It is composed of 3 linkages (1, 2, and 3), and 4 noncoplanar joints. It is parameterized by its angular deviation *α*, its characteristic length *d* and its angular orientation *η*. The coordinates frames are also represented. Its associated DH table is given in Table 3.

Considering now each leg (*A*, *B*, and *C*) of the mechanism, it is possible to parametrize the configuration of $C_{ee}$ also by following the associated serial kinematic chain, e.g., using the Denavit-Hartenberg (DH) convention (Siciliano and Khatib, 2007). Note first that the three legs share the same kinematic structure, which is shown in Figure 4. As such, they can all be parametrized with the same DH table given in Table 3. Note that, by the construction of the system, linkages 1 and 3 are identical, and *d* and *α* are fixed parameters that define the shared legs geometry, while *η* is different for each leg.

**TABLE 3:** Denavit-Hartenberg parameters of a generic leg.

#	$q_{i}$	$d_{i}$	$a_{i}$	$\alpha_{i}$
0	0	0	0	*η*
1	$q_{1}$	0	0	$\frac{\pi }{2}$
2	$q_{2}$	d	0	*α*
3	$q_{3}$	-d	0	$\frac{\pi }{2}$
4	$q_{4}$	0	0	π−η

Calling $\left(q_{1},q_{2},q_{3},q_{4}\right)$ and *η* the joint angles and the base angle of the generic leg, the global transformation matrix going from the system base to the end effector through the leg Tlee0 is defined as

Tlee0=Tl110Tl22Tl33Tl44Tlee. (6)

where all the intermediate matrices are defined based on the parameters in Table 3. After calculations, we obtain:

Tlee0=(T11T12T13T14T21T22T23T24T31T32T33T340001), (7)

where all the non-trivial terms are defined in Supplementary Appendix A.

### 3.2 Closing the Kinematic Chain

When the joint is mounted, i.e., the legs are assembled, the assembly constrains the final frame associated to each leg (and the generic frame Tee0) to be equal, i.e.,

Tee0=TAee0=TBee0=TCee0. (8)

As it is well-known, the solution of Eq. 8, is fundamental to yield both the direct and inverse kinematics of the parallel system. An important aid to facilitate this comes from Sofka et al., (2006a), where it was found that a feasible mounting of each leg holds for

$$\{\begin{array}{l} q_{4}=-q_{1}\\ q_{2}=q_{3}+\pi \end{array}\;\;\text{i}.\text{e}.,\;\;\{\begin{array}{l} c_{4}=c_{1}\\ s_{4}=-s_{1}\\ c_{3}=-c_{2}\\ s_{3}=-s_{2}. \end{array}$$ (9)

Therefore, after simplifications, Eq. 7 can be written as

$$\begin{array}{l} T_{11}=-g_{1}\left(q_{1},q_{2},\alpha \right)\\ T_{21}=-s_{\eta }g_{2}\left(q_{1},q_{2},\alpha \right)+c_{\eta }g_{3}\left(q_{1},q_{2},\alpha \right)\\ T_{31}=s_{\eta }g_{3}\left(q_{1},q_{2},\alpha \right)+c_{\eta }g_{2}\left(q_{1},q_{2},\alpha \right)\\ T_{12}=s_{\eta }g_{2}\left(q_{1},q_{2},\alpha \right)-c_{\eta }g_{3}\left(q_{1},q_{2},\alpha \right)\\ T_{22}=-2c_{\eta }s_{\eta }g_{4}\left(q_{1},q_{2},\alpha \right)+c_{\eta }^{2}g_{5}\left(q_{1},q_{2},\alpha \right)+s_{\eta }^{2}g_{6}\left(q_{2},\alpha \right)\\ T_{32}=c_{\eta }s_{\eta }g_{7}\left(q_{1},q_{2},\alpha \right)+\left(c_{\eta }^{2}-s_{\eta }^{2}\right)g_{4}\left(q_{1},q_{2},\alpha \right)\\ T_{13}=-c_{\eta }g_{2}\left(q_{1},q_{2},\alpha \right)-s_{\eta }g_{3}\left(q_{1},q_{2},\alpha \right)\\ T_{23}=c_{\eta }s_{\eta }g_{7}\left(q_{1},q_{2},\alpha \right)+\left(c_{\eta }^{2}-s_{\eta }^{2}\right)g_{4}\left(q_{1},q_{2},\alpha \right)\\ T_{33}=-2c_{\eta }s_{\eta }g_{4}\left(q_{1},q_{2},\alpha \right)+s_{\eta }^{2}g_{5}\left(q_{1},q_{2},\alpha \right)+c_{\eta }^{2}g_{6}\left(q_{2},\alpha \right)\\ T_{14}=-dh_{1}\left(q_{1},q_{2},\alpha \right)\\ T_{24}=-d\left[c_{\eta }h_{2}\left(q_{1},q_{2},\alpha \right)+s_{\eta }h_{3}\left(q_{2},\alpha \right)\right]\\ T_{34}=d\left[c_{\eta }h_{3}\left(q_{2},\alpha \right)-s_{\eta }h_{2}\left(q_{1},q_{2},\alpha \right)\right], \end{array}$$ (10)

where is a family of functions defined as

$$\left\{ \begin{array}{l} g_{1}\left(q_{1},q_{2},\alpha \right)=c_{1}^{2}\left(c_{2}^{2}-s_{2}^{2}c_{\alpha }\right)+s_{1}^{2}c_{\alpha }+2c_{1}s_{1}s_{2}s_{\alpha }\\ g_{2}\left(q_{1},q_{2},\alpha \right)=c_{2}\left[s_{1}s_{\alpha }-c_{1}s_{2}\left(1+c_{\alpha }\right)\right]\\ g_{3}\left(q_{1},q_{2},\alpha \right)=\left(c_{1}^{2}-s_{1}^{2}\right)s_{2}s_{\alpha }+c_{1}s_{1}\left[2c_{\alpha }-c_{2}^{2}\left(1+c_{\alpha }\right)\right]\\ g_{4}\left(q_{1},q_{2},\alpha \right)=c_{2}\left[c_{1}s_{\alpha }+s_{1}s_{2}\left(1+c_{\alpha }\right)\right]\\ g_{5}\left(q_{1},q_{2},\alpha \right)=c_{1}^{2}c_{\alpha }+s_{1}^{2}\left(c_{2}^{2}-s_{2}^{2}c_{\alpha }\right)-2c_{1}s_{1}s_{2}s_{\alpha }\\ g_{6}\left(q_{2},\alpha \right)=s_{2}^{2}-c_{2}^{2}c_{\alpha }\\ g_{7}\left(q_{1},q_{2},\alpha \right)=\left(c_{2}^{2}-s_{2}^{2}\right)\left(1+c_{\alpha }\right)+c_{1}^{2}\left[s_{2}^{2}\left(1+c_{\alpha }\right)-\left(1-c_{\alpha }\right)\right]-2c_{1}s_{1}s_{2}s_{\alpha }, \end{array}\right.$$ (11)

and ${\left(h_{i}\right)}_{i\in \left\{1,2,3\right\}}$ is a family of functions defined as

$$\{\begin{array}{l} h_{1}\left(q_{1},q_{2},\alpha \right)=c_{1}s_{2}s_{\alpha }-s_{1}\left(1-c_{\alpha }\right)\\ h_{2}\left(q_{1},q_{2},\alpha \right)=c_{1}\left(1-c_{\alpha }\right)+s_{1}s_{2}s_{\alpha }\\ h_{3}\left(q_{2},\alpha \right)=c_{2}s_{\alpha }. \end{array}$$ (12)

The families ${\left(g_{i}\right)}_{i\in \left\{1,\ldots ,7\right\}}$ and ${\left(h_{i}\right)}_{i\in \left\{1,2,3\right\}}$ are introduced to improve the readability of the matrices and the calculations.

As expected from Sofka et al. (2006a), and as we will confirm with our derivation, the assembled system has 2 DoF. Thus, it is possible to parametrize the position and orientation of the reachable end effector configurations with only two variables. For convenience we choose

$$u={\left(\begin{array}{cc} \alpha_{y} & \alpha_{z} \end{array}\right)}^{T}.$$ (13)

To fully characterize the set of feasible end effector configurations it is necessary to specify the other 4 variables in Eq. 3.

To derive the angular orientation $\alpha_{x}$ as a function of u, a key point relies on noting that

Tlee0(3,2)=Tlee0(2,3), (14)

and then, by using Eq. 5 and rearranging the terms, it is possible to extract $\alpha_{x}$ as

$$\alpha_{x}=\text{arctan}\left(\frac{s_{y}s_{z}}{c_{y}+c_{z}}\right),$$ (15)

a result in agreement with (Sofka et al., 2006a).

Then, the analysis can be extended by deriving an explicit formulation of the position of the center of the end effector $\left(x_{ee},y_{ee},z_{ee}\right)$ as a function of u. Only the key results are given in the following part and the reader is invited to refer to Supplementary Appendix B for more detailed proofs of the different assumed expressions.Based on Eqs 5 and 8, we have

xee=Tlee0(1,4) (16a)

yee=Tlee0(2,4) (16b)

zee=Tlee0(3,4), (16c)

from which it can be shown [based on (10)] that

$$x_{ee}^{2}+y_{ee}^{2}+z_{ee}^{2}=2d^{2}\left(1-c_{\alpha }\right),$$ (17)

which gives the distance square of the point $O_{ee}$ to the point $O_{0}$. This distance is thus independent of the positions of the legs as it depends only on *d* and *α* which are two parameters inherent of the mechanical design of the system and could also be found based on a geometric closed form of the system.

Defining such distance as

$$L:=d\sqrt{2\left(1-c_{\alpha }\right)},$$ (18)

and using the previous equations, it can be shown that

$$x_{ee}^{2}=L^{2}\left(\frac{1+c_{y}c_{z}}{2}\right)$$ (19a)

$${\left(y_{ee}+z_{ee}\right)}^{2}=\frac{L^{2}}{2\left(1+c_{y}c_{z}\right)}{\left(s_{y}-c_{y}s_{z}\right)}^{2}$$ (19b)

$${\left(y_{ee}-z_{ee}\right)}^{2}=\frac{L^{2}}{2\left(1+c_{y}c_{z}\right)}{\left(s_{y}+c_{y}s_{z}\right)}^{2}.$$ (19c)

According to the convention defined previously, we have

$$\forall \left(\alpha_{y},\alpha_{z}\right)\in \left(-\pi /2;\pi /2\right),\;x_{ee}\geq 0,$$

therefore, using Eq. 19a, we obtain

$$x_{ee}=L\sqrt{\frac{1+c_{y}c_{z}}{2}}.$$ (20)

Then, the system defined by Eqs. 19b and 19c has 4 pairs $\left(y_{ee},z_{ee}\right)$ of solutions. Yet, according to the convention previously defined, we know that $y_{ee}$ should increase when $\alpha_{z}$ is increasing and $z_{ee}$ should decrease when $\alpha_{y}$ is increasing. Therefore, there is only one suitable solution for the pair $\left(y_{ee},z_{ee}\right)$, which is

$$\{\begin{array}{l} y_{ee}=L\frac{c_{y}s_{z}}{\sqrt{2\left(1+c_{y}c_{z}\right)}}\\ z_{ee}=-L\frac{s_{y}}{\sqrt{2\left(1+c_{y}c_{z}\right)}}. \end{array}$$ (21)

To conclude, the proposed system has 2 DoF in orientation and the explicit formulation of the general mapping *T* is as

$$\left(\begin{array}{c} \alpha_{x}\\ \alpha_{y}\\ \alpha_{z}\\ x_{ee}\\ y_{ee}\\ z_{ee} \end{array}\right)=\left[\begin{array}{c} \arctan\left(\frac{s_{y}s_{z}}{c_{y}+c_{z}}\right)\\ \alpha_{y}\\ \alpha_{z}\\ L\sqrt{\frac{1+c_{y}c_{z}}{2}}\\ L\frac{c_{y}s_{z}}{\sqrt{2\left(1+c_{y}c_{z}\right)}}\\ -L\frac{s_{y}}{\sqrt{2\left(1+c_{y}c_{z}\right)}} \end{array}\right]=T\left(\alpha_{y},\alpha_{z}\right).$$ (22)

This mapping is always defined for all $\left(\alpha_{y},\alpha_{z}\right)$ in (−π/2;π/2).

### 3.3 Inverse Kinematics Model

This subsection aims to find the position of all the joints of each leg of the system as a function of the pose of the end effector, x.

Based on the chosen parametrization, calculations for each leg are very similar. Therefore, only generic calculations are presented in the following section. Moreover, using the reduction of parametrization, presented in Eq. 9, the aim is equivalent to find only $q_{1}$ and $q_{2}$ as functions of x. The reader is invited to refer to Supplementary Appendix C for a more detailed demonstration of the following equations.

Firstly, to obtain $q_{2}$, the idea is to isolate $h_{3}\left(q_{2},\alpha \right)$ defined in Eq. 12. It can be shown that $c_{2}$ is defined by the equation

$$c_{2}=\frac{1}{ds_{\alpha }}\left(c_{\eta }z_{ee}-s_{\eta }y_{ee}\right).$$ (23)

From Eq. 23, it is possible to extract $q_{2}$ as a function of x and the parameters of the system, as follows:

$$q_{2}=\text{arccos}\left[\frac{1}{ds_{\alpha }}\left(c_{\eta }z_{ee}-s_{\eta }y_{ee}\right)\right].$$ (24)

Besides that, it can be shown that $c_{1}$ and $s_{1}$ should satisfy the following system

$$\left(\begin{array}{c} s_{1}\\ c_{1} \end{array}\right)=\frac{1}{\tilde{d}}\left(\begin{array}{cc} 1-c_{\alpha } & s_{2}s_{\alpha }\\ -s_{2}s_{\alpha } & 1-c_{\alpha } \end{array}\right)\left(\begin{array}{c} x_{ee}\\ -\left(c_{\eta }y_{ee}+s_{\eta }z_{ee}\right) \end{array}\right),$$ (25)

where $\tilde{d}$ is defined as

$$\tilde{d}=d\left[{\left(1-c_{\alpha }\right)}^{2}+{\left(s_{2}s_{\alpha }\right)}^{2}\right].$$ (26)

As $q_{2}$ is known in Eq. 24, it is possible to extract from Eq. 25 $q_{1}$ as a function of only x and the parameters of the system, as follows:

$$q_{1}=\text{arctan}\left[\frac{-s_{2}s_{\alpha }\left(c_{\eta }y_{ee}+s_{\eta }z_{ee}\right)-\left(c_{\alpha }-1\right)x_{ee}}{\left(c_{\alpha }-1\right)\left(c_{\eta }y_{ee}+s_{\eta }z_{ee}\right)-s_{2}s_{\alpha }x_{ee}}\right].$$ (27)

The complete solution for a generic leg is then

$$\begin{array}{l} q_{1}=\text{arctan}\left[\frac{-s_{2}s_{\alpha }\left(c_{\eta }y_{ee}+s_{\eta }z_{ee}\right)-\left(c_{\alpha }-1\right)x_{ee}}{\left(c_{\alpha }-1\right)\left(c_{\eta }y_{ee}+s_{\eta }z_{ee}\right)-s_{2}s_{\alpha }x_{ee}}\right]\\ q_{2}=\text{arccos}\left[\frac{1}{ds_{\alpha }}\left(c_{\eta }z_{ee}-s_{\eta }y_{ee}\right)\right]\\ q_{3}=\text{arccos}\left[\frac{1}{ds_{\alpha }}\left(c_{\eta }z_{ee}-s_{\eta }y_{ee}\right)\right]-\pi \\ q_{4}=-\text{arctan}\left[\frac{-s_{2}s_{\alpha }\left(c_{\eta }y_{ee}+s_{\eta }z_{ee}\right)-\left(c_{\alpha }-1\right)x_{ee}}{\left(c_{\alpha }-1\right)\left(c_{\eta }y_{ee}+s_{\eta }z_{ee}\right)-s_{2}s_{\alpha }x_{ee}}\right]. \end{array}$$ (28)

It can be easily adapted for each leg, by replacing the generic parameters and variables with the proper ones. For instance, by replacing in the previous equations, $\left(q_{1},q_{2},q_{3},q_{4}\right)$ with $\left(q_{A1},q_{A2},q_{A3},q_{A4}\right)$ and *η* with $\eta_{A}$, it is possible to obtain the inverse kinematics of leg *A*, namely ${\text{T}}_{A_{ee}}$, and analogously for legs *B* and *C*.

### 3.4 Differential Kinematics

The explicit inverse kinematics model is given as

$$q_{act}=f_{IK}\left(u\right),$$ (29)

where $q_{act}$ represents the vector of the actuated joints of the system, defined as

$$q_{act}={\left(\begin{array}{ccc} q_{A1} & q_{B1} & q_{C1} \end{array}\right)}^{T},$$ (30)

the function $f_{IK}$ can be written as

$$f_{IK}\left(u\right)=\left(\begin{array}{c} f_{IK,A}\left(u\right)\\ f_{IK,B}\left(u\right)\\ f_{IK,C}\left(u\right) \end{array}\right),$$ (31)

and u stands for the minimal parametrization of the system, defined in Eq. 13.By differentiation of Eq. 29, we obtain

$${\dot{q}}_{act}=A_{u}\dot{u},$$ (32)

where the matrix $A_{u}$ is defined as

$$A_{u}=\frac{\partial f_{IK}}{\partial u}\left(u\right)=\left(\begin{array}{cc} \partial_{\alpha_{y}}f_{IK,A}\left(u\right) & \partial_{\alpha_{z}}f_{IK,A}\left(u\right)\\ \partial_{\alpha_{y}}f_{IK,B}\left(u\right) & \partial_{\alpha_{z}}f_{IK,B}\left(u\right)\\ \partial_{\alpha_{y}}f_{IK,C}\left(u\right) & \partial_{\alpha_{z}}f_{IK,C}\left(u\right) \end{array}\right),$$ (33)

where for any function *f* and variable $x_{i}$, $\partial_{x_{i}}f$ stands for the partial derivative of *f* with respect to $x_{i}$. An explicit formulation of $A_{u}$ can be computed based on the results presented in Section 3.3 and is reported in Supplementary Appendix D. This formulation depends explicitly on the pose of the end effector.

### 3.5 Static Equilibrium

Using the kinetostatic duality, we obtain

$$\tau_{u}=A_{u}^{T}\tau_{act},$$ (34)

where $\tau_{u}$ in ${\mathbb{R}}^{2}$ represents the torques of the end effector associated with u and $\tau_{act}$ stands for the torques of the actuated joints, i.e., it could be denoted as

$$\tau_{act}={\left(\begin{array}{ccc} \tau_{A1} & \tau_{B1} & \tau_{C1} \end{array}\right)}^{T}.$$ (35)

For a given external wrench **f**, acting on the end effector, the static equilibrium gives us, using Eq. 34, the relationship between the external wrench **f** and the torque of the actuated joints as

$$f=A_{u}^{T}\tau_{act}.$$ (36)

As we assume to be working outside of the singularities, the rank of the matrix $A_{u}$ is 2, so it has a pseudo-inverse. So, it is possible to derive from Eq. 36, the torque $\tau_{act}^{\text{*}}$ required to balance a given external wrench $f^{\text{*}}$ acting on the end effector, as

$$\tau_{act}^{\text{*}}=A_{u}{\left(A_{u}^{T}A_{u}\right)}^{-1}f^{\text{*}}+\lambda N_{0},$$ (37)

where *λ* is a scalar real and $N_{0}$ represents a basis of the solutions of

$$A_{u}^{T}X=0.$$ (38)

The reader is invited to refer to Section 4.1 for details on the calculation of $N_{0}$.

### 3.6 Static Stiffness

To define the static stiffness, we first need to define $\tau_{act}$ as a function of the torque of the elastic elements.

Define $f_{S_{A}},f_{S_{B}}f_{S_{C}}$ the torque functions of the nonlinear elastic elements placed on each leg of the system. Letting *∈{A,B,C}, $f_{S_{\text{*}}}$ represents thus the torque of the actuated joint on the leg * and is a function of the deflection $\delta_{\text{*}}$ of the elastic mechanism defined as

$$\delta_{\text{*}}=q_{\text{*}1}-\theta_{M_{\text{*}}},$$ (39)

where $\theta_{M_{\text{*}}}$ denotes the position of each motor $M_{\text{*}}$. $\theta_{M_{A}},\theta_{M_{B}}$ and $\theta_{M_{C}}$ are the controlled input variables of the proposed system.

Therefore, $\tau_{act}$ can be written as

$$\tau_{act}\left(\delta \right)=\left(\begin{array}{c} f_{S_{A}}\left(q_{A1}-\theta_{M_{A}}\right)\\ f_{S_{B}}\left(q_{B1}-\theta_{M_{B}}\right)\\ f_{S_{C}}\left(q_{C1}-\theta_{M_{C}}\right) \end{array}\right)=\left(\begin{array}{c} f_{S_{A}}\left(\delta_{A}\right)\\ f_{S_{B}}\left(\delta_{B}\right)\\ f_{S_{C}}\left(\delta_{C}\right) \end{array}\right),$$ (40)

where δ stands for the vector of the deflections of the elastic elements defined as

$$\delta ={\left(\begin{array}{ccc} \delta_{A} & \delta_{B} & \delta_{C} \end{array}\right)}^{T}.$$ (41)

Let’s define $\sigma_{ee}$, the static stiffness of the end effector as

$$\sigma_{ee}=\frac{\partial \tau_{u}}{\partial u}.$$ (42)

Using the chain rule we have that

$$\sigma_{ee}=\frac{\partial \tau_{u}}{\partial \tau_{act}}\frac{\partial \tau_{act}}{\partial q_{act}}\frac{\partial q_{act}}{\partial u}.$$ (43)

Based on Eq. 34, we have

$$\frac{\partial \tau_{u}}{\partial \tau_{act}}=A_{u}^{T}$$ (44)

and based on Eqs. 29 and 33, we have

$$\frac{\partial q_{act}}{\partial u}=A_{u}.$$ (45)

Moreover, we have

$$\frac{\partial \tau_{act}}{\partial q_{act}}=K\left(\delta \right),$$ (46)

where K(δ) is a diagonal matrix in ${\mathbb{R}}^{3\times 3}$ with the stiffness of each leg on its diagonal, defined as

$$K\left(\delta \right)=\left(\begin{array}{ccc} \frac{\partial f_{S_{A}}}{\partial q_{A1}}\left(\delta_{A}\right) & 0 & 0\\ 0 & \frac{\partial f_{S_{B}}}{\partial q_{B1}}\left(\delta_{B}\right) & 0\\ 0 & 0 & \frac{\partial f_{S_{C}}}{\partial q_{C1}}\left(\delta_{C}\right) \end{array}\right).$$ (47)

Therefore, we obtain

$$\sigma_{ee}=A_{u}^{T}K\left(\delta \right)A_{u}.$$ (48)

### 3.7 Cartesian Compliance and Cost Function

To represent the stiffness characteristics of the system, we decide to represent in the space its Cartesian compliance, the quantity denoted by c in ${\mathbb{R}}^{3\times 3}$ such that

dp=cdf, (49)

where *p* in ${\mathbb{R}}^{3}$ is a point attached to the end effector and f in ${\mathbb{R}}^{3}$ is the Cartesian external forces applied at this point, i.e., *f* represents only the forces applied on p in the directions *x*, *y* and *z*. This decision is motivated by the fact that, as shown in the previous section, the end-effector of the system has only 2 Dof, which means that the system will not allow motions in one of the three Cartesian directions of space, thus in that same direction the stiffness would be infinite. Therefore its inverse (i.e., the compliance) will be more manageable mathematically, and easier to represent graphically.

According to the minimal parametrization defined in Eq. 13, *p* is a function of u. So, we have

dp=Jdu, (50)

where the matrix J is in ${\mathbb{R}}^{3\times 2}$ and denotes the Jacobian matrix of the function p with respect to the variables of u.

$$\tau_{u}=J^{T}f$$ (51)

where $\tau_{u}$ denotes the forces associated with *u* and f stands for the Cartesian forces of *p*, from which we obtain in the static equilibrium

$$d\tau_{u}=J^{T}df.$$ (52)

Moreover, using the definition of the static stiffness of the end effector in Eq. 42, we obtain

$$d\tau_{u}=\sigma_{ee}du.$$ (53)

The matrix K is always invertible and as previously said, the rank of the matrix $A_{u}$ is 2, as we assume to be working outside of the singularities, so it has a pseudo-inverse. Therefore, based on Eq. 48, $\sigma_{ee}$ is invertible. So,

$$du=\sigma_{ee}^{-1}d\tau_{u}$$ (54)

with

$$\sigma_{ee}^{-1}={\left(A_{u}^{T}A_{u}\right)}^{-1}A_{u}^{T}K^{-1}A_{u}{\left(A_{u}^{T}A_{u}\right)}^{-1}.$$ (55)

So, by injecting Eq. 52 in Eq. 54, we obtain

$$du=\sigma_{ee}^{-1}J^{T}df,$$ (56)

which gives us, based on Eq. 50, the expression of the Cartesian compliance c such as

$$dp=cdf\;\text{with}\;c=J\sigma_{ee}^{-1}J^{T}.$$ (57)

Knowing the compliance matrix, it is possible to derive the cost function used in the optimization algorithm such as

$$y=\sum_{i=1}^{n_{targets}}\sum_{j=2}^{3}\sum_{k=2}^{j}{\left[\frac{c\left(i,j\right)-c_{ref}\left(i,j\right)}{c_{ref}\left(i,j\right)}\right]}^{2},$$ (58)

where $c_{ref}$ stands for the desired compliance matrix based on the definition of the desired behavior and $n_{targets}$ the number of desired compliant behaviors. As a note, the sum is only done on the lower-diagonal and diagonal components (and non-null) of the compliances matrices as they are symmetric. The minimization problem is done using MATLAB, based on the sqp-algorithm.

### 3.8 Graphical Representation Methodology

To get a visual representation of the Cartesian compliance, we plot the ellipsoid associated with the matrix *c*. This ellipsoid is defined using the singular value decomposition of *c*. Its axes are directed by the eigenvectors of the matrix and their half lengths are defined by their respective associated eigenvalue. The ellipsoid is centered in *p*. Hence, the ellipsoid represents the possible deflection of the system in the space for a normalized external wrench, and thus the greater the ellipsoid is, the more compliant is the system.

**FIGURE 5:**
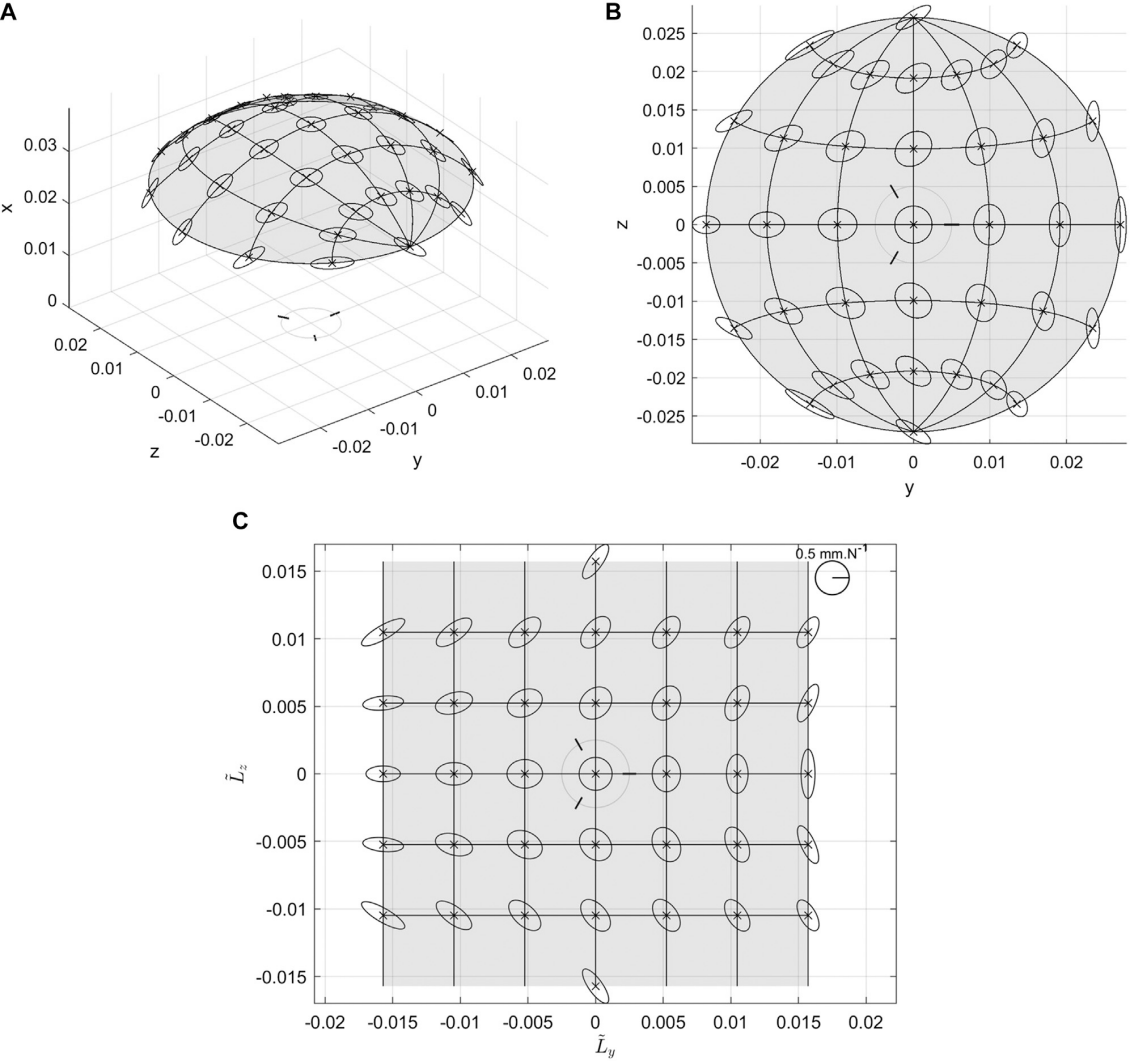
Cartesian compliance ellipsoids of the system **(A)** 3D view, **(B)** top view and **(C)** flattened (see text for detail) representations. Simulations are made at the center of the end effector, with $\eta_{A}$, $\eta_{B}$ and $\eta_{C}$ evenly distributed around the basis, α=π/4, d=50 mm and c = (1 rad/Nm, 1 rad/Nm, 1 rad/Nm)

Figure 5 shows an example of the various representations and views of the compliance ellipsoids for a specific configuration of the system. One way to represent the compliance ellipsoids is to draw them in 3 dimensions. They can be viewed either from a 3D perspective, such as in Figure 5A, or from a top view of the system [i.e., on a projection on the plane (*y*, *z*)] such as in Figure 5B. A second option is a representation in 2D, where the same ellipsoids of the Cartesian compliance are plotted in a plane $\left({\tilde{L}}_{y},{\tilde{L}}_{z}\right)$, such as in Figure 5C. This plane corresponds to an arbitrary scaled distance between the points while following the surface of all of the reachable points. It represents therefore the ellipsoids as if they were always seen from the normal of every point. Note that these ellipsoids are not defined in the $\left({\tilde{L}}_{y},{\tilde{L}}_{z}\right)$ plane, so not to be affected by the representation singularities associated with the selected parametrization in ${\tilde{L}}_{z}=\pm \pi /2$. On the contrary, they are defined in the tangent plane to each point of the curved surface in Figure 5A. Note also that for ${\tilde{L}}_{z}=\pm \pi /2$ the ellipsoids are arbitrarily oriented choosing ${\tilde{L}}_{x}=0$. Note that we will preferentially use this representation in the rest of the paper, referring to it as *flattened representation*.

The analogous of meridians and parallels have been plotted also to help the understanding of the corresponding ellipses in the example figures. In each representation, the locus of all the possible points reachable of *p* is also plotted as a colored area even if the ellipsoids are displayed only in some discrete points. This area is referred to as the workspace of the system. The flattened representation has the advantage not to distort the ellipsoids and not to be sensitive to the Cartesian workspace which may be affected by the design parameters of the mechanism. So, this representation will mainly be used thereafter. Finally, the angular orientation of the axes of the first joint of each leg is plotted as small black lines in the different graphs.

Besides, one thing to notice is that the ellipsoids are only ellipses, tangent to the workspace. This is in agreement with the consideration made at the beginning of this subsection, which motivated the choice of representing the system compliance instead of its stiffness.

## 4 Results

### 4.1 Variable Stiffness Ability of the System

In this section, we prove that it is possible to have a decoupled control of the position and the stiffness of the system, i.e., that it is possible to modify the spring load without moving the end effector.

Let us consider first the static stiffness given in Eq. 48 and show that it can be modulated. For a given position u, the matrix $A_{u}$ is decided. Moreover, in case of the elastic elements are nonlinear, the matrix K depends on the deflection δ which can be controlled through the position of the motors $\theta_{M_{\text{*}}}$. Therefore, the matrix K and thus the static stiffness of the system $\sigma_{ee}$ can be controlled using nonlinear elastic elements in series of each kinematic leg. This result agrees with the one in case of the design of one DoF physically compliant VSA using the antagonistic approach, as reported in the literature (Vanderborght et al., 2013).

Similarly, the Cartesian compliance of the system defined in Eq. 57 can be controlled if the elastic elements are nonlinear by modifying the positions of the motors $\theta_{M_{\text{*}}}$.

Let us prove now that the compliance can be modulated at a fixed position. The first step is to derive a possible expression of $N_{0}$, a basis of the space of the solutions of Eq. 38. The matrix $A_{u}$, defined in Eq. 33 can be written as

$$A_{u}=\left(\begin{array}{cc} Q_{y} & Q_{z} \end{array}\right),$$ (59)

where $Q_{y}$ and $Q_{z}$ stand respectively for the first and second columns of matrix $A_{u}$.

Therefore, *X* in ${\mathbb{R}}^{3}$ is a solution of Eq. 38 if and only if

$$\{\begin{array}{l} Q_{y}^{T}X=0\\ Q_{z}^{T}X=0 \end{array}.$$ (60)

This system of equations has a space of solution of dimension 1 if and only if $Q_{y}$ and $Q_{z}$, i.e., the two columns of $A_{u}$, are non-collinear which can be written as $det\left(A_{u}^{T}A_{u}\right)\neq 0$. This is always true as we assume to be working outside of the mechanical singularities, i.e., the rank of $A_{u}$ is equal to 2.

Therefore, the Solutions of Eq. 38 are described as

$$\left\{X\in {\mathbb{R}}^{3}|\exists \lambda \in \mathbb{R},\;X=\lambda N_{0}\right\}.$$ (61)

A possible definition of $N_{0}$ is thus

$$N_{0}=\frac{Q_{y}\times Q_{z}}{∥Q_{y}\times Q_{z}{∥}_{2}},$$ (62)

where .×. and $∥.{∥}_{2}$ denote respectively the cross product and the euclidean norm of vectors in ${\mathbb{R}}^{3}$.

Therefore, along the space described by $N_{0}$, we have a one dimensional space where $\tau_{act}$ is varying without affecting the equilibrium position of the end effector. The matrix $A_{u}$ is thus constant as the pose of the end effector is decided and therefore, $q_{act}$ is fixed. Assuming the non-linearity of the elastic elements, there is a space to control the compliance of the end effector without modifying the pose of the end effector. This is a one-dimensional space and can be therefore used to control a one-dimensional variable linked to the stiffness, such as the sum of the potential elastic energies stored in each elastic elements.

**FIGURE 6:**
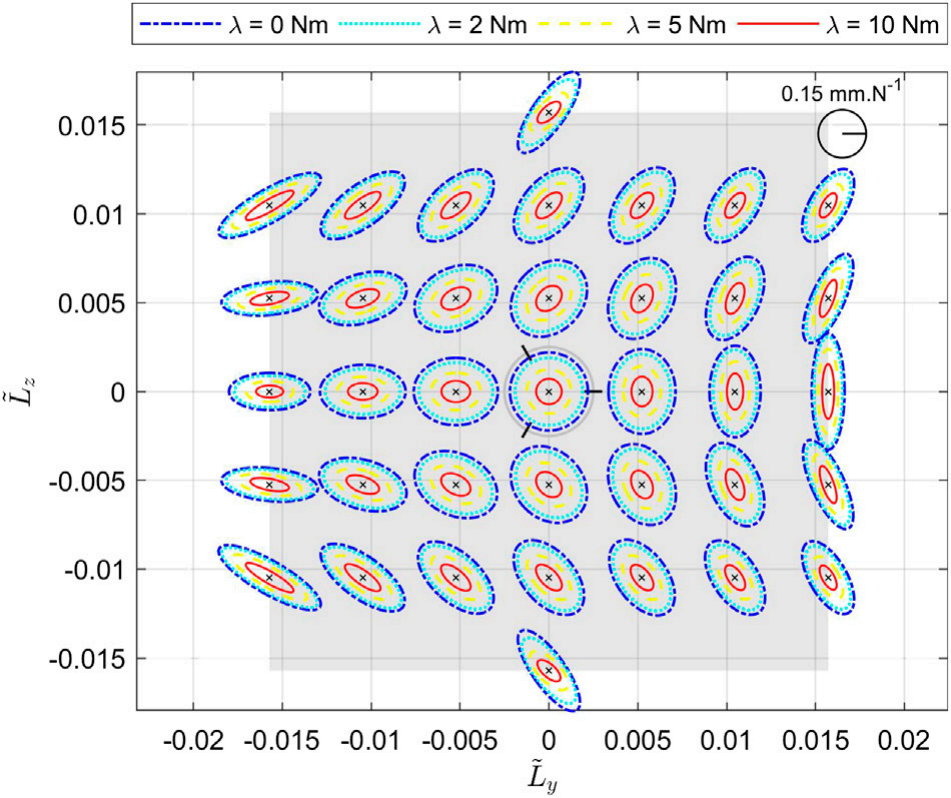
System Compliance in flattened representation, as a consequence of the internal modulation while *λ* is varying in the set {0 Nm, 2 Nm, 5 Nm, 10 Nm}. Simulations are made at the center of the end effector, with $\eta_{A}$, $\eta_{B}$ and $\eta_{C}$ evenly distributed around the basis, α=π/4, d=50 mm, and all the elastic elements identical ($K_{\text{*}}=1\;\text{Nm}/\text{rad}$ and $\delta_{0_{\text{*}}}=1\;\text{rad}$).

The effect of the internal modulation is studied while varying the parameter *λ* defined in Eq. 37, with the model of the elastic elements defined in Eq. 1. It has to be noticed that with this definition of $f_{S_{\text{*}}}$, the stiffness of each elastic element is an even function in *λ*, as $f_{S_{\text{*}}}$ is an odd function in *λ*, using Eq. 37. Figure 6 represents the Cartesian compliance ellipses for various values^1^ of *λ*. We can see how, acting on *λ*, the ellipses scale homogeneously in all directions, without appreciable changes in shape and orientation like the co-contraction of human muscles (Milner, 2002; Perreault et al., 2002).

**FIGURE 7:**
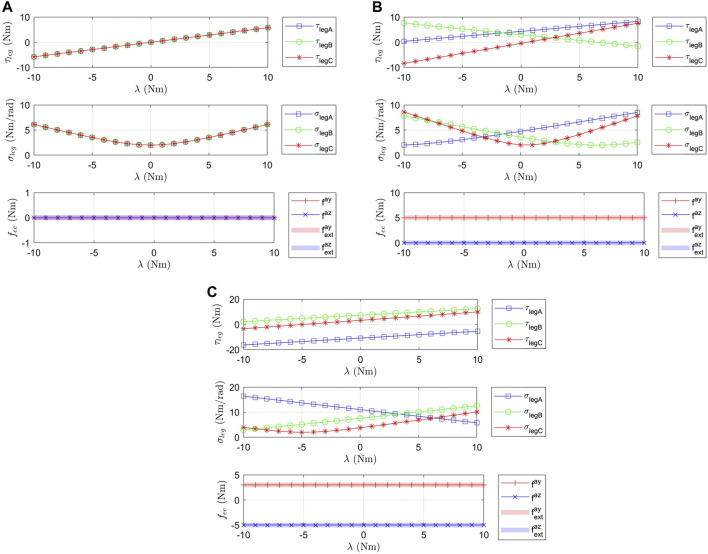
Evolution of the torque of each leg $\tau_{leg}$, their respective stiffness $\sigma_{leg}$ and the force balanced by the end effector $\left(f^{ay},f^{az}\right)$ (in the directions of *α*_*y*_ and *α*_*z*_) for *λ* varying between -10 Nm and 10 Nm and for various equilibrium positions $u_{eq}$ and external forces $f_{eq}=\left(f_{ext}^{ay},f_{ext}^{az}\right)$. **(A)** corresponds to $u_{eq}$ = (0, 0) deg − $f_{eq}$ = (0, 0) Nm, **(B)** to $u_{eq}$ = (90, 0) deg − $f_{eq}$ = (5, 0) Nm and **(C)** to $u_{eq}$ = (60,30) deg − $f_{eq}$ = (3, −5) Nm.

Moreover, Figure 7 shows the evolution of the legs torque and stiffness, and the output force at three different positions of the end effector, highlighting how the actuation of *λ* changes the stiffness but does not change the external equilibrium. Indeed, the external wrench balanced at the end effector $\left(f^{ay},f^{az}\right)$ is not modified whereas the stiffness of each leg is affected and thus the stiffness of the end effector.

As a final note, it is possible to show that the stiffness of the system is increasing when the external wrench is increasing. In addition, although the stiffness increases slightly in all directions, the increase is larger in the direction of the applied external force. For sake of space, the associated figures are not displayed here as this is an expected behavior for a VSA (Vanderborght et al., 2013).

### 4.2 General Design Guidelines

In this section, we give the results of the effects of the geometric parameters on the compliance of the system according to the methodology described in Section 2.3. To show their effects, the Cartesian compliance ellipsoids are plotted at the center of the end effector (i.e., *p* in the previous analysis is $O_{ee}$) for several positions of the end effector [parametrized by $u={\left(\begin{array}{cc} \alpha_{y} & \alpha_{z} \end{array}\right)}^{T}$] and various geometries defined by the design parameters. When it is relevant, the workspace of the system is also plotted as a colored area even if the ellipsoids are displayed only in some discrete points.

**FIGURE 8:**
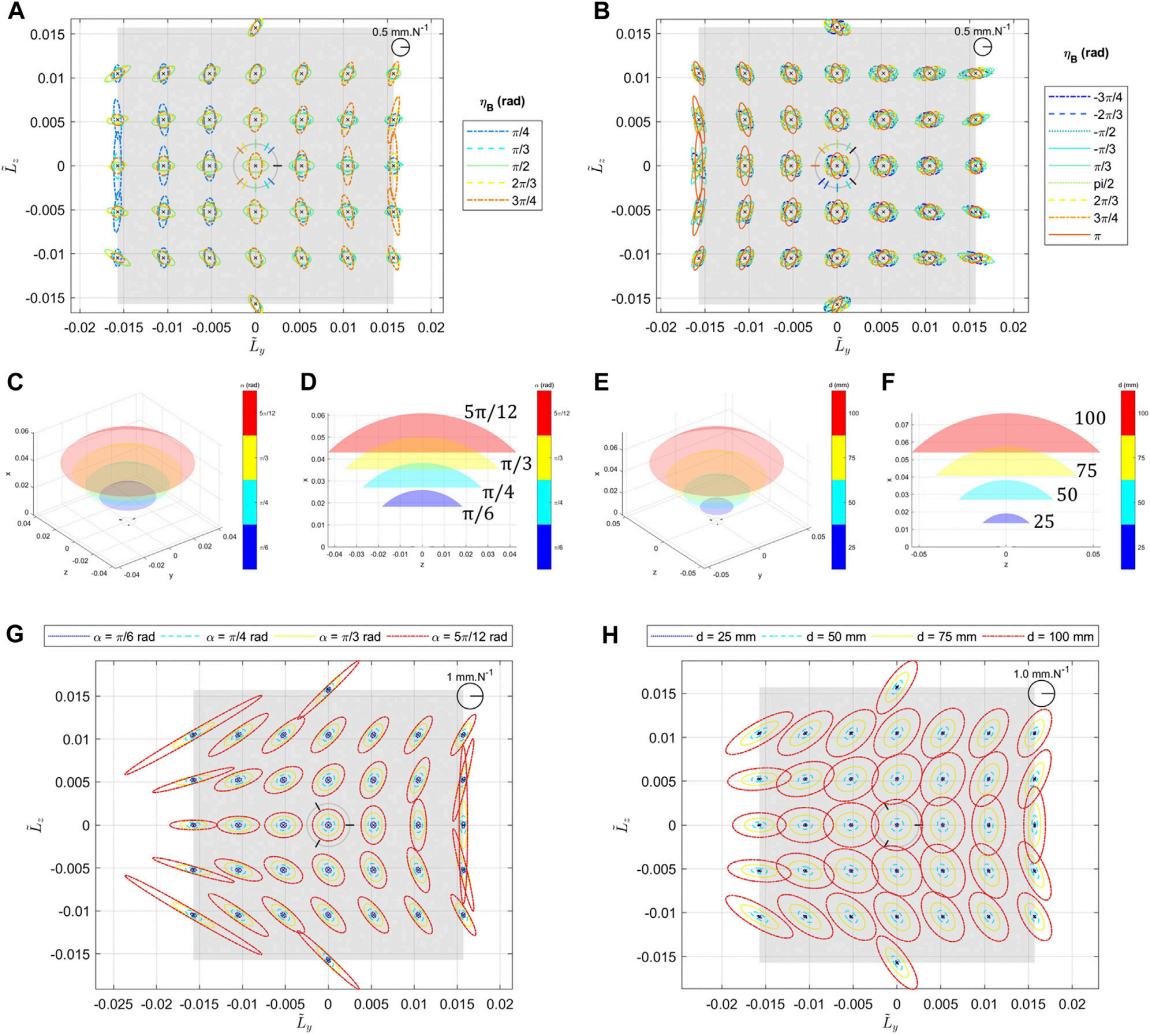
Effects of the geometric parameters on the system compliance. **(A)** represents the system compliance in flattened representation, as the angular positions of the two legs $\eta_{B}$ and $\eta_{C}$ vary in a symmetric way. $\eta_{A}$ is fixed and equal to 0, $\eta_{C}=-\eta_{B}$ and $\eta_{B}$ is varying in the set {π/4, π/3, π/2, 2π/3, 3π/4}. Simulations are made at the center of the end effector, with α=π/4, d=50 mm and *c* = (1 rad/Nm 1 rad/Nm 1 rad/Nm). **(B)** represents the system compliance in flattened representation, when the angular position of one leg $\left(\eta_{B}\right)$ varies in an asymmetric way. $\eta_{A}$ and $\eta_{C}$ are fixed and respectively equal to π/4 and −π/4 and $\eta_{B}$ is varying in the set {−3π/4, −2π/3, −π/2, −π/3, π/3, π/2, 2π/3, 3π/4, *π*}. Simulations are made at the center of the end effector, with α=π/4, d=50 mm and c = (1 rad/Nm, 1 rad/Nm, 1 rad/Nm). **(C)** and **(D)** represent the workspace of the system in 3D view and side view when *α* is varying in the set {π/6, π/4, π/3, 5π/12}. **(E)** is the associated flattened representation of the compliance ellipsoids. Simulations are made at the center of the end effector, with $\eta_{A}$, $\eta_{B}$ and $\eta_{C}$ evenly distributed around the basis, d=50 mm and c = (1 rad/Nm, 1 rad/Nm, 1 rad/Nm). **(F)** and **(G)** represent the workspace of the system in 3D view and side view when *d* is varying in the set {25 mm, 50 mm, 75 mm, and 100 mm}. **(H)** is the associated flattened representation of the compliance ellipsoids. Simulations are made at the center of the end effector, with $\eta_{A}$, $\eta_{B}$ and $\eta_{C}$ evenly distributed around the basis, α=π/4 and c = (1 rad/Nm, 1 rad/Nm, 1 rad/Nm).

Figure 8 represents the different figures of the simulations when the various design parameters are varying. Figures 8A,B show the results when the angular positions of the legs are varying in a symmetric or asymmetric way. We can see that in both cases, depending on the angular position, it is possible to stretch the ellipses in one of its two main directions by modifying the value of the angle and to rotate them. Figures 8C–E show the effects of *α* on the Cartesian compliance ellipses and the workspace. We can notice that the compliance ellipses are globally increasing when *α* is increasing. Figures 8F–H show the effects of *d* on the Cartesian compliance ellipses and the workspace. We can notice that the compliance ellipses are globally increasing when *d* is increasing.

As it is shown in the different figures, all the design parameters do not have the same effect nor the same intensity on the compliance characteristic of the system. For instance, the effects of *α* are more important in terms of stretching the ellipses, especially at the borders of the workspace, whereas *d* has more a global influence everywhere that can be compensating by using different elastic elements. Moreover, it is important to notice that both previous results go together with a stretch of the surface of the feasible positions of the end effector, shown in Figures 8C,D,F,G. This explains that the increase in compliance is also an effect of the increase of the lever arm of the forces. Combining the previous two effects, it is conceivable to change the shape of the ellipses while maintaining the feasible surface and ellipses area roughly constant by changing alpha and d simultaneously in opposite directions. However, it appears that *α* and *d* are not enough to modulate the ratio of the ellipse at (0,0) position where we observe always a circle for an even distribution of the legs as shown in Figures 8E,H. This shows a limitation of using only these two parameters to modify the embedded behavior in the system and justify the use of the angular positions of the legs as geometric parameters.

### 4.3 Design Parameters for Human-Like Joints

**FIGURE 9:**
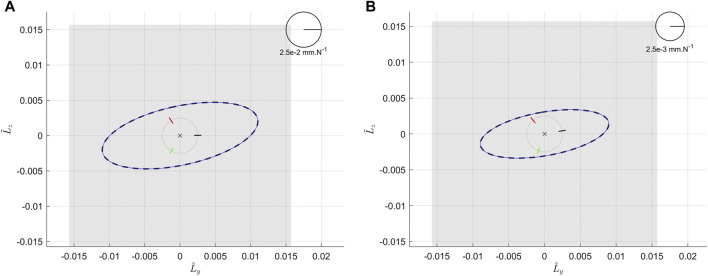
**(A)** Compliance ellipses at the neutral position in absence of external load for a wrist joint. The dashed black line stands for the targeted ellipse extracted from human biomechanical data in Pando et al. (2014). The blue line represents compliant behavior of the proposed system with an even distribution of the legs around the basis. **(B)** Compliance ellipses at the neutral position in absence of external load for an ankle joint. The dashed black line stands for the targeted ellipse extracted from human biomechanical data in Lee et al. (2014)). The blue line represents compliant behaviour of the proposed system with an even distribution of the legs around the basis.

In this section, we derive sets of parameters to match the passive compliant behavior of a human wrist (Pando et al., 2014) and a human ankle (Lee et al., 2014). In both cases, the idea is to find the best set of parameters to match the targeted ellipse at the neutral position, defined in Section 2.4.1. Regarding the input of the algorithm, we would like to match 3 characteristics. Therefore, with 12 design parameters the solutions are not unique. We can then set additional constraints besides the realistic values of the design parameters. As we are considering passive behavior, we set *λ* equal to 0. This means that in absence of external load, no motor torque is required to get the passive behavior of the system. Moreover, we consider an even distribution of the legs around the basis. Figure 9 represents solutions for a wrist and an ankle considering theses constraints. Their associated design parameters are reported in Table 4. Therefore, it is possible to get feasible systems based on the proposed architecture with a passive compliant behavior similar to the human wrist or a human ankle.

**TABLE 4:** Design parameters to match human wrist and human ankle.

	Human wrist	Human ankle
$\eta_{A}$ (°)	1	10
$\eta_{B}$ (°)	121	130
$\eta_{C}$ (°)	241	250
*α* (°)	45	45
*d* (mm)	50	50
$K_{1}$ (Nm/rad)	0.939	0.479
$K_{2}$ (Nm/rad)	1.968	1.514
$K_{3}$ (Nm/rad)	1.990	1.959
$\delta_{0,1}$ (rad^−1^)	1.53	4.47
$\delta_{0,2}$ (rad^−1^)	0.28	0.02
$\delta_{0,3}$ (rad^−1^)	0.44	0.03
*λ* (Nm)	0	0

## 5 Discussion

In this work, we proposed and studied a new concept to design a 2 DoF VSA. To reduce the complexity of the mechanical implementation and increase the compactness of the system, we use only 3 motors. This is the minimum number of motors to be able to have a 2 DoF mechanism fully actuated in positions and partially actuated in stiffness. Therefore, our solution is more compact than most of the solutions existing in the current state-of-the-art (refer for instance to the works of (Grebenstein et al., 2011; Romano et al., 2014; Weckx et al., 2014; Koganezawa, 2017; Tan et al., 2017)). To the best knowledge of the authors, only the work in (Stoeffler et al., 2018) follows the same approach of using only 3 motors.

Obviously, a limitation of our proposed solution relies in the loss of DoF in the control of stiffness of the system with respect to the existing 2 DoF VSAs. Indeed, our system can control the overall size of its endpoint stiffness ellipse but not its stiffness independently in the two directions of motion. Yet this approach is motivated by the fact that human beings modulate their stiffness through both their posture (exploiting the geometry and properties of their limbs) and co-contraction (Milner, 2002; Perreault et al., 2002). More interestingly, their effects are quite distinct as the limb geometry and posture have a large impact on the shape and orientation of the ellipse and the co-contraction on the overall size (Perreault et al., 2002). Therefore, by implementing physically the human joint geometry and allowing the control of the overall size of the stiffness, we can design human-like joints with the potential of natural control of both the position and stiffness. To the best knowledge of the authors, there is no other work following this approach.

However, the challenge of the mechanical implementation of the passive compliance of human joints remains. That is why we derived a model of our system and studied the effect of its main design parameters on the characteristics of the compliance ellipses. Additionally to the guidelines given in Section 4.2, it appears that the influences of the angular positions of the leg are interesting as they allow us to stretch or rotate the compliance ellipses in certain directions. Combining all of the effects, it will be possible to embed some specific behavior in the design for a specified application. And indeed, we derived sets of design parameters to match the passive behavior of a human wrist and a human ankle.

**FIGURE 10:**
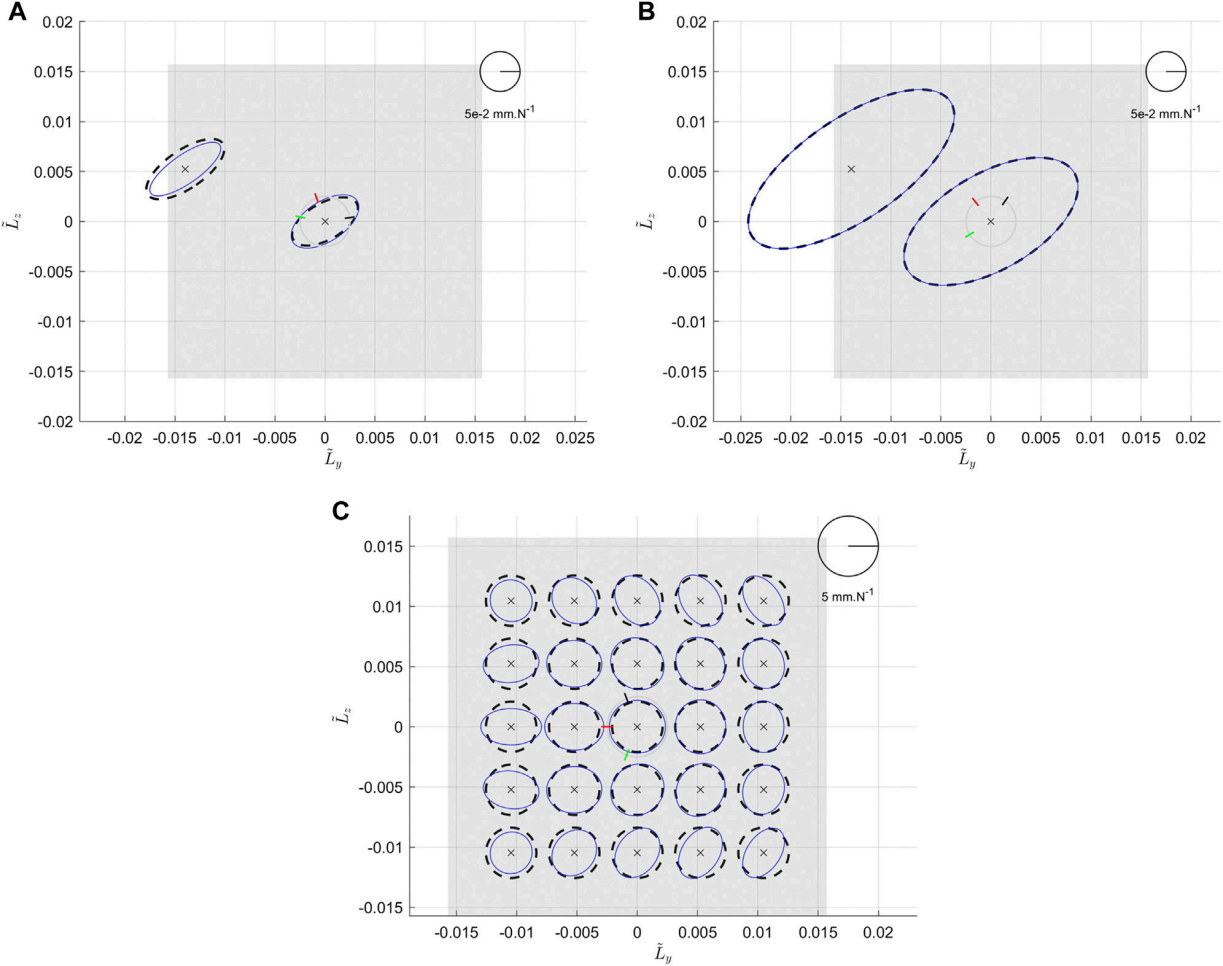
**(A)** (case 1.1) represents the compliance ellipses at the neutral position in absence of external load with *λ* null. **(B)** (case 1.2) represents the compliance ellipses at the neutral position in absence of external load without any additional constraints. **(C)** (case 2.1) represents the compliance ellipses in a large portion of the workspace. In all figures the dashed black lines stand for the targeted ellipses and the blue lines stand for the solutions.

For now, biomechanical data exists only at the neutral position of the joints. However, in the future, additional data could be available on various positions of the workspace of the joints. In this case, we may want to match several ellipses. In the proposed system, it is possible to increase the number of desired ellipses. Figures 10A,B present solutions for 2 desired ellipses. The case 1.1 corresponds to a solution with an additional constraint of the vector *λ* equal to 0. As it is shown, this solution may be insufficient. Case 1.2 stands for a solution without any additional constraints. In this case, the two ellipses are well matched. It shows that the system could match additional passive behaviors, for instance by relaxing the constraint of passivity, previously explained (*λ* equal to 0). The associated design parameters of these 2 cases are given in Table 5.

**TABLE 5:** Design parameters of cases 1.1 to 2.1.

	Case 1.1	Case 1.2	Case 2.1
$\eta_{A}$ (°)	9	55	111
$\eta_{B}$ (°)	110	128	180
$\eta_{C}$ (°)	170	211	249
*α* (°)	30	49	40
*d* (mm)	49	49	10
$K_{1}$ (Nm/rad)	2.000	0.789	0.01
$K_{2}$ (Nm/rad)	2.000	1.986	0.01
$K_{3}$ (Nm/rad)	0.01	1.051	0.01
$\delta_{0,1}$ (rad^−1^)	1.08	1.79	6.50
$\delta_{0,2}$ (rad^−1^)	0.64	0.63	4.86
$\delta_{0,3}$ (rad^−1^)	6.5	3.20	6.50
*λ* (Nm)	[0 0]	[5.94 7.9e^−5^]	**0** ^25^

To go further, we could also apply the proposed concept to more complex joints (such as the shoulder or the hip). Unfortunately, such biomechanical data are not yet available. We are more than aware that this is not an easy task and we can only encourage the biomechanical scientific community to pursue their current efforts in this direction, such as the work proposed in Hunt and Lee (2019).

As another further step, we could imagine having a lot of desired ellipses. The case 2.1 for instance corresponds to an isotropic compliant behavior of the system in a large portion of the workspace. Such a system could be conceived for applications using teleoperated robots where we would like to have the same behavior over the workspace. Concretely, the required behavior is now a compliance circle over 25 positions over a grid of 5 × 5 positions between −60° and 60° in both directions. In this case, we have thus 75 characteristics to match. Similar results are obtained if the vector^2^ *λ* is free and if *λ* is set to 0, ensuring thus the passivity of the compliant behavior. Therefore, only the results when *λ* is null, noted **0** ^25^, are displayed here. Figure 10C represents the result of the optimization algorithm and the parameters are given in Table 5. As it is shown in Figure 10C, the behavior is not exactly matched but it seems acceptable over a smaller range of the workspace.

Obviously, in the case of several desired ellipses, there are some mechanical limitations but we can refer to Section 4.2 to get the general trends and to design useful desired behavior. And it seems possible to design artificial joints that match the passive compliant behavior of human articulations in various positions of the workspace, to investigate the potential of such features in robotic systems.

Another limitation of this study is that we did not explore the space of all the possible spring characteristics. Indeed, we used a model (referred to as hyperbolic sine springs) that we already implemented in previous works (Catalano et al., 2011; Lemerle et al., 2019), as similar elastic behaviors can reproduce some attributes of an antagonistic pair of muscles driving a joint (Garabini et al., 2017). However, to improve the human-likeliness of the output stiffness characteristic, we could study more extensively on the best nonlinear spring characteristic that should be implemented. This was out of the intended scope of this work, but future studies could investigate further on this topic using additional biomechanical data to implement human-like stiffness behaviors.

## 6 Conclusion

This paper presents the concept of a new configurable 2 DoF variable stiffness joint. The system is based on redundant parallel nonlinear elastic actuation, using the antagonistic approach inspired by the human musculoskeletal system. The kinematic structure of the system is based on the architecture of the Omni-Wrist III, designed by Rosheim et al. and we propose to add in series nonlinear elastic systems to obtain a VSA. To get a compact design, only three motors are used and we prove that this allows us to control both the position and the overall stiffness of system. A one-dimensional quantity linked to the stiffness can thus be controlled like the voluntary co-contraction of human muscles. Moreover, we outline the impact of the main design parameters on the compliant behavior of the system as general guidelines. We describe an optimization methodology to fine-tune the mechanical implementation of this type of system to match the specific passive behavior of a human wrist and ankle.

We believe that the proposed design could find many applications, including industrial manipulators and prosthetic devices for various joints, due to its versatility. The sets of parameters obtained to match the passive behavior of a human wrist are particularly relevant as they open the possibility to design a compact system with an embedded passive behavior close to the human one. A new prototype of a compact physically compliant variable stiffness wrist is currently under development.

## Data Availability

The original contributions presented in the study are included in the article/Supplementary Material, further inquiries can be directed to the corresponding author.

## Footnotes

1
*λ* is only varying in the positive values as the stiffness of each elastic element is an even function in *λ* in case of a hyperbolic sine spring.

2 In this example, the vector *λ* is in ${\mathbb{R}}^{25}$.
