# Supplementary material for: A Configurable Architecture for Two Degree-of-Freedom Variable Stiffness Actuators to Match the Compliant Behavior of Human Joints
Source: Front Robot AI. 2021 Mar 12;8:614145. doi: 10.3389/frobt.2021.614145 (PMC8006398; doi:10.3389/frobt.2021.614145)
Supplement: Supplementary file 1 [file datasheet1.pdf]

APPENDIX A

The appendix details the non-trivial components of the global transformation matrix defined in Section 3.1 as

$$ {}^{0}\mathrm{T}_{l_{ee}} = \begin{pmatrix} T_{11} & T_{12} & T_{13} & T_{14} \\ T_{21} & T_{22} & T_{23} & T_{24} \\ T_{31} & T_{32} & T_{33} & T_{34} \\ 0 & 0 & 0 & 1 \end{pmatrix} . \tag{7} $$

They are equal to

$$ \begin{aligned} T_{11} &= c_1c_4(c_2c_3 - s_2s_3c_\alpha) + c_1s_4s_2s_\alpha + s_1c_4s_3s_\alpha + s_1s_4c_\alpha \\ T_{21} &= c_\eta[-c_1c_4s_3s_\alpha - c_1s_4c_\alpha + s_1c_4(c_2c_3 - s_2s_3c_\alpha + s_1s_4s_2s_\alpha] - s_\eta[c_4(s_2c_3 + c_2s_3c_\alpha) - s_4c_2s_\alpha] \\ T_{31} &= c_\eta[c_4(s_2c_3 + c_2s_3c_\alpha) - s_4c_2s_\alpha] + s_\eta[-c_1c_4s_3s_\alpha - c_1s_4c_\alpha + s_1c_4(c_2c_3 - s_2s_3c_\alpha) + s_1s_4s_2s_\alpha] \\ T_{12} &= s_\eta[c_1(c_2s_3 + s_2c_3c_\alpha) - s_1c_3s_\alpha] + c_\eta[-c_1c_4s_2s_\alpha + s_1s_4s_3s_\alpha + c_1s_4(c_2c_3 - s_2s_3c_\alpha) - s_1c_4c_\alpha] \\ T_{22} &= c_\eta s_\eta[c_1c_3s_\alpha + s_1(c_2s_3 + s_2c_3c_\alpha) - c_4c_2s_\alpha - s_4(s_2c_3 + c_2s_3c_\alpha)] - s_\eta^2[s_2s_3 - c_2c_3c_\alpha] \\ &\quad +c_\eta^2[c_1c_4c_\alpha - (c_1s_4s_3 + s_1c_4s_2)s_\alpha + s_1s_4(c_2c_3 - s_2s_3c_\alpha)] \\ T_{32} &= c_\eta s_\eta[s_2s_3 - c_2c_3c_\alpha - (c_1s_4s_3 + s_1c_4s_2)s_\alpha + c_1c_4c_\alpha + s_1s_4(c_2c_3 - s_2s_3c_\alpha)] \\ &\quad +s_\eta^2[s_1(c_2s_3 + s_2c_3c_\alpha) + c_1c_3s_\alpha] + c_\eta^2[c_2c_4s_\alpha + s_4(s_2c_3 + c_2s_3c_\alpha)] \\ T_{13} &= s_\eta[-c_1c_4s_2s_\alpha + s_1s_4s_3s_\alpha + c_1s_4(c_2c_3 - s_2s_3c_\alpha) - s_1c_4c_\alpha] - c_\eta[c_1(c_2s_3 + s_2c_3c_\alpha) - s_1c_3s_\alpha] \\ T_{23} &= c_\eta s_\eta[s_2s_3 - c_2c_3c_\alpha - (c_1s_4s_3 + s_1c_4s_2)s_\alpha + c_1c_4c_\alpha + s_1s_4(c_2c_3 - s_2s_3c_\alpha)] \\ &\quad -c_\eta^2[s_1(c_2s_3 + s_2c_3c_\alpha) + c_1c_3s_\alpha] - s_\eta^2[c_2c_4s_\alpha + s_4(s_2c_3 + c_2s_3c_\alpha)] \\ T_{33} &= c_\eta s_\eta[c_4c_2s_\alpha + s_4(s_2c_3 + c_2s_3c_\alpha) - c_1c_3s_\alpha - s_1(c_2s_3 + s_2c_3c_\alpha)] - c_\eta^2[s_2s_3 - c_2c_3c_\alpha] \\ &\quad +s_\eta^2[c_1c_4c_\alpha - (c_1s_4s_3 + s_1c_4s_2)s_\alpha + s_1s_4(c_2c_3 - s_2s_3c_\alpha)] \\ T_{14} &= -d[c_1s_2s_\alpha - s_1(1 - c_\alpha)] \\ T_{24} &= -d[c_\eta(c_1(1 - c_\alpha) + s_1s_2s_\alpha) + s_\eta c_2s_\alpha] \\ T_{34} &= d[c_\eta c_2s_\alpha - s_\eta(c_1(1 - c_\alpha) + s_1s_2s_\alpha)], \end{aligned} \tag{63} $$

where c_η, s_η, c_α, and s_α stand respectively for $\cos(\eta)$, $\sin(\eta)$, $\cos(\alpha)$, and $\sin(\alpha)$. And for all i in {1,2,3,4}, c_i stands for $\cos(q_i)$ and s_i for $\sin(q_i)$. Replacing, in the previous equations, (q_1, q_2, q_3, q_4) with $(q_{A1}, q_{A2}, q_{A3}, q_{A4})$ and η with η_A, it is possible to obtain the forward kinematics of leg A, namely $\mathrm{T}_{A_{ee}}$, and analogously for legs B and C.

APPENDIX B

In this appendix, we prove the following set of expressions used to derive the general mapping in Section 3.2:

$$x_{ee}^2 + y_{ee}^2 + z_{ee}^2 = 2d^2(1 - c_\alpha) \tag{64a}$$

$$x_{ee}^2 = L^2\left(\frac{1 + c_y c_z}{2}\right) \tag{64b}$$

$$(y_{ee} + z_{ee})^2 = \frac{L^2}{2(1 + c_y c_z)}(s_y - c_y s_z)^2 \tag{64c}$$

$$(y_{ee} - z_{ee})^2 = \frac{L^2}{2(1 + c_y c_z)}(s_y + c_y s_z)^2. \tag{64d}$$

The proofs rely on different equations extracted from Sections 3.1 and 3.2, which are referred with their associated numbering in the main text.

We first focus on the proof of (64a). Using the following equations

$$\begin{aligned} x_{ee} &= {}^0\mathrm{T}_{l_{ee}}(1,4) \\ y_{ee} &= {}^0\mathrm{T}_{l_{ee}}(2,4) \\ z_{ee} &= {}^0\mathrm{T}_{l_{ee}}(3,4), \end{aligned} \tag{16}$$

and the simplified formulation of ${}^0\mathrm{T}_{l_{ee}}$ provided in the following equation

$$\begin{aligned}
T_{11} &= -g_1(q_1, q_2, \alpha) \\
T_{21} &= -s_\eta g_2(q_1, q_2, \alpha) + c_\eta g_3(q_1, q_2, \alpha) \\
T_{31} &= s_\eta g_3(q_1, q_2, \alpha) + c_\eta g_2(q_1, q_2, \alpha) \\
T_{12} &= s_\eta g_2(q_1, q_2, \alpha) - c_\eta g_3(q_1, q_2, \alpha) \\
T_{22} &= -2c_\eta s_\eta g_4(q_1, q_2, \alpha) + c_\eta^2 g_5(q_1, q_2, \alpha) + s_\eta^2 g_6(q_2, \alpha) \\
T_{32} &= c_\eta s_\eta g_7(q_1, q_2, \alpha) + (c_\eta^2 - s_\eta^2) g_4(q_1, q_2, \alpha) \\
T_{13} &= -c_\eta g_2(q_1, q_2, \alpha) - s_\eta g_3(q_1, q_2, \alpha) \\
T_{23} &= c_\eta s_\eta g_7(q_1, q_2, \alpha) + (c_\eta^2 - s_\eta^2) g_4(q_1, q_2, \alpha) \\
T_{33} &= -2c_\eta s_\eta g_4(q_1, q_2, \alpha) + s_\eta^2 g_5(q_1, q_2, \alpha) + c_\eta^2 g_6(q_2, \alpha) \\
T_{14} &= -d h_1(q_1, q_2, \alpha) \\
T_{24} &= -d(c_\eta h_2(q_1, q_2, \alpha) + s_\eta h_3(q_2, \alpha)) \\
T_{34} &= d(c_\eta h_3(q_2, \alpha) - s_\eta h_2(q_1, q_2, \alpha)),
\end{aligned} \tag{10}$$

it is possible to state that

$$\begin{cases} x_{ee} &= -dh_1 \\ y_{ee} &= -d(c_\eta h_2 + s_\eta h_3) \\ z_{ee} &= d(c_\eta h_3 - s_\eta h_2) \end{cases}. \tag{65}$$

Therefore, the squared value of each components is

$$\begin{cases} x_{ee}^2 &= d^2 h_1^2 \\ y_{ee}^2 &= d^2(c_\eta^2 h_2^2 + s_\eta^2 h_3^2 + 2c_\eta s_\eta h_2 h_3) \\ z_{ee}^2 &= d^2(s_\eta^2 h_2^2 + c_\eta^2 h_3^2 - 2c_\eta s_\eta h_2 h_3) \end{cases}. \tag{66}$$

So, we obtain

$$x_{ee}^2 + y_{ee}^2 + z_{ee}^2 = d^2[h_1^2 + h_2^2 + h_3^2]. \tag{67}$$

In addition, based on the definition of the functions $(h_i)_{i\in\{1,2,3\}}$ as

$$\begin{cases} h_1(q_1, q_2, \alpha) &= c_1 s_2 s_\alpha - s_1(1 - c_\alpha) \\ h_2(q_1, q_2, \alpha) &= c_1(1 - c_\alpha) + s_1 s_2 s_\alpha \\ h_3(q_2, \alpha) &= c_2 s_\alpha, \end{cases} \tag{12}$$

we have[1]

$$\begin{cases} h_1^2 &= (c_1 s_2)^2 s_\alpha^2 + s_1^2(1 - c_\alpha)^2 - 2c_1 s_1 s_2 s_\alpha(1 - c_\alpha) \\ &= (1 - c_\alpha)[(c_1 s_2)^2(1 + c_\alpha) + s_1^2(1 - c_\alpha) - 2c_1 s_1 s_2 s_\alpha] \\ h_2^2 &= c_1^2(1 - c_\alpha)^2 + (s_1 s_2)^2 s_\alpha^2 + 2c_1 s_1 s_2 s_\alpha(1 - c_\alpha) \\ &= (1 - c_\alpha)[c_1^2(1 - c_\alpha) + (s_1 s_2)^2(1 + c_\alpha) + 2c_1 s_1 s_2 s_\alpha] \\ h_3^2 &= c_2^2 s_\alpha^2 \\ &= (1 - c_\alpha)(1 + c_\alpha)c_2^2 \end{cases}. \tag{68}$$

Therefore, we obtain

$$h_1^2 + h_2^2 + h_3^2 = (1 - c_\alpha)[1 - c_\alpha + s_2^2(1 + c_\alpha) + c_2^2(1 + c_\alpha)]. \tag{69}$$

So, considering that $s_2^2 + c_2^2 = 1$, we obtain

$$h_1^2 + h_2^2 + h_3^2 = 2(1 - c_\alpha). \tag{70}$$

Therefore, by injecting (70) in (67), we obtain the desired expression

$$x_{ee}^2 + y_{ee}^2 + z_{ee}^2 = 2d^2(1 - c_\alpha). \tag{71}$$

[1] As a preliminary remark, we should notice that $s_\alpha^2 = 1 - c_\alpha^2 = (1 - c_\alpha)(1 + c_\alpha)$.

Let us prove now (64b). From (66), we have

$$x_{ee}^2 = d^2 h_1^2, \tag{72}$$

which can be written, by adding 0, as

$$x_{ee}^2 = d^2(h_1^2 + h_2^2 + h_3^2) - d^2(h_2^2 + h_3^2). \tag{73}$$

The sum $h_1^2 + h_2^2 + h_3^2$ is already known in (70). The sum $h_2^2 + h_3^2$ can be derived as follows. Based on (68), we have

$$h_2^2 + h_3^2 = (1 - c_\alpha)[c_1^2(1 - c_\alpha) + (s_1 s_2)^2(1 + c_\alpha) + 2c_1 s_1 s_2 s_\alpha + (1 + c_\alpha)c_2^2]. \tag{74}$$

By using $s_1^2 = 1 - c_1^2$ and $s_2^2 + c_2^2 = 1$, (74) is equivalent to

$$h_2^2 + h_3^2 = (1 - c_\alpha)[c_1^2(1 - c_\alpha - s_2^2(1 + c_\alpha)) + (1 + c_\alpha) + 2c_1 s_1 s_2 s_\alpha], \tag{75}$$

which can be converted into

$$\begin{aligned} h_2^2 + h_3^2 &= (1 - c_\alpha)[1 + c_1^2(1 - s_2^2 - s_2^2 c_\alpha) - c_1^2 c_\alpha + c_\alpha + 2c_1 s_1 s_2 s_\alpha] \\ &= (1 - c_\alpha)[1 + c_1^2(c_2^2 - s_2^2 c_\alpha) + (1 - c_1^2)c_\alpha + 2c_1 s_1 s_2 s_\alpha] \\ &= (1 - c_\alpha)[1 + c_1^2(c_2^2 - s_2^2 c_\alpha) + s_1^2 c_\alpha + 2c_1 s_1 s_2 s_\alpha]. \end{aligned} \tag{76}$$

Therefore, based on the definition of g_1 in

$$\begin{cases} g_1(q_1, q_2, \alpha) &= c_1^2(c_2^2 - s_2^2 c_\alpha) + s_1^2 c_\alpha + 2c_1 s_1 s_2 s_\alpha \\ g_2(q_1, q_2, \alpha) &= c_2(s_1 s_\alpha - c_1 s_2(1 + c_\alpha)) \\ g_3(q_1, q_2, \alpha) &= (c_1^2 - s_1^2)s_2 s_\alpha + c_1 s_1(2c_\alpha - c_2^2(1 + c_\alpha)) \\ g_4(q_1, q_2, \alpha) &= c_2(c_1 s_\alpha + s_1 s_2(1 + c_\alpha)) \\ g_5(q_1, q_2, \alpha) &= c_1^2 c_\alpha + s_1^2(c_2^2 - s_2^2 c_\alpha) - 2c_1 s_1 s_2 s_\alpha \\ g_6(q_2, \alpha) &= s_2^2 - c_2^2 c_\alpha \\ g_7(q_1, q_2, \alpha) &= (c_2^2 - s_2^2)(1 + c_\alpha) + c_1^2(s_2^2(1 + c_\alpha) - (1 - c_\alpha)) - 2c_1 s_1 s_2 s_\alpha, \end{cases} \tag{11}$$

we obtain

$$h_2^2 + h_3^2 = (1 - c_\alpha)(1 + g_1). \tag{77}$$

By injecting (77) into (73), we obtain

$$x_{ee}^2 = 2d^2(1 - c_\alpha) - d^2(1 - c_\alpha)(1 + g_1). \tag{78}$$

Using the definition of L given Section 3.2 as

$$L := d\sqrt{2(1 - c_\alpha)}, \tag{18}$$

(78) can be written as

$$x_{ee}^2 = L^2(\frac{1-g_1}{2}). \tag{79}$$

Moreover, based on the following equality of the transformation matrices given in Section 3.2

$$^0\mathrm{T}_{ee} = {}^0\mathrm{T}_{A_{ee}} = {}^0\mathrm{T}_{B_{ee}} = {}^0\mathrm{T}_{C_{ee}}, \tag{8}$$

we have

$$^0\mathrm{T}_{ee}(1,1) = {}^0\mathrm{T}_{l_{ee}}(1,1), \tag{80}$$

Based on the respective definitions of the matrices $^0\mathrm{T}_{ee}$ and $^0\mathrm{T}_{l_{ee}}$ given in

$$^0\mathrm{T}_{ee} = \begin{pmatrix} c_zc_y & c_zs_xs_y - s_zc_x & c_zs_yc_x + s_zs_x & x_{ee} \\ s_zc_y & s_zs_xs_y + c_zc_x & s_zs_yc_x - c_zs_x & y_{ee} \\ -s_y & s_xc_y & c_xc_y & z_{ee} \\ 0 & 0 & 0 & 1 \end{pmatrix}, \tag{5}$$

and in (10), (80) gives

$$g_1 = -c_yc_z. \tag{81}$$

Therefore, we conclude that

$$x_{ee}^2 = L^2\left(\frac{1+c_yc_z}{2}\right). \tag{82}$$

Finally, let us prove (64c) and (64d) as the calculations are very similar. Based on (65) we have

$$\begin{aligned} (y_{ee}+z_{ee})^2 &= d^2(h_2^2+h_3^2) + 2d^2(c_\eta s_\eta(h_2^2-h_3^2) - (c_\eta^2 - s_\eta^2)h_2h_3) \\ (y_{ee}-z_{ee})^2 &= d^2(h_2^2+h_3^2) - 2d^2(c_\eta s_\eta(h_2^2-h_3^2) - (c_\eta^2 - s_\eta^2)h_2h_3). \end{aligned} \tag{83}$$

The sum $h_2^2 + h_3^2$ is already known in (77). The difference $h_2^2 - h_3^2$ and the product h_2h_3 can be computed following similar calculations as before. Based on (68), we have

$$\begin{aligned} h_2^2 - h_3^2 &= (1-c_\alpha)[c_1^2(1-c_\alpha) + (s_1s_2)^2(1+c_\alpha) + 2c_1s_1s_2s_\alpha - (1+c_\alpha)c_2^2] \\ &= (1-c_\alpha)[c_1^2(1-c_\alpha) + (1-c_1^2)s_2^2(1+c_\alpha) - c_2^2(1+c_\alpha) + 2c_1s_1s_2s_\alpha] \\ &= (1-c_\alpha)[c_1^2(1-c_\alpha - s_2^2(1+c_\alpha) - (c_2^2-s_2^2)(1+c_\alpha) + 2c_1s_1s_2s_\alpha] \\ &= -(1-c_\alpha)[c_1^2(s_2^2(1+c_\alpha - (1-c_\alpha)) + (c_2^2-s_2^2)(1+c_\alpha) - 2c_1s_1s_2s_\alpha] \end{aligned} \tag{84}$$

and

$$\begin{aligned} h_2h_3 &= (c_1(1-c_\alpha) + s_1s_2s_\alpha)c_2s_\alpha \\ &= c_2(c_1(1-c_\alpha)s_\alpha + s_1s_2s_\alpha^2) \\ &= c_2(1-c_\alpha)(c_1s_\alpha + s_1s_2(1+c_\alpha)). \end{aligned} \tag{85}$$

Therefore, based on the definitions of g_4 and g_7 in (11), we obtain

$$h_2^2 - h_3^2 = -(1 - c_\alpha)g_7 \tag{86a}$$

$$h_2 h_3 = (1 - c_\alpha)g_4. \tag{86b}$$

So, we obtain

$$\begin{array}{rcl} (y_{ee} + z_{ee})^2 & = & d^2(1 - c_\alpha)(1 + g_1) - 2d^2(1 - c_\alpha)(c_\eta s_\eta g_7 + (c_\eta^2 - s_\eta^2)g_4) \\ (y_{ee} - z_{ee})^2 & = & d^2(1 - c_\alpha)(1 + g_1) + 2d^2(1 - c_\alpha)(c_\eta s_\eta g_7 + (c_\eta^2 - s_\eta^2)g_4). \end{array} \tag{87}$$

Moreover, based on (8), we have

$$\begin{array}{rcl} {}^0\mathrm{T}_{ee}(1,1) & = & {}^0\mathrm{T}_{l_{ee}}(1,1) \\ {}^0\mathrm{T}_{ee}(3,2) & = & {}^0\mathrm{T}_{l_{ee}}(3,2), \end{array} \tag{88}$$

which gives, based on the respective definitions of the matrices ${}^0\mathrm{T}_{ee}$ and ${}^0\mathrm{T}_{l_{ee}}$ given in (5) and (10),

$$\begin{array}{rcl} g_1 & = & -c_y c_z \\ c_\eta s_\eta g_7 + (c_\eta^2 - s_\eta^2)g_4 & = & s_x c_y. \end{array} \tag{89}$$

Therefore, we obtain

$$\begin{array}{rcl} (y_{ee} + z_{ee})^2 & = & d^2(1 - c_\alpha)(1 - c_y c_z - 2s_x c_y) \\ (y_{ee} - z_{ee})^2 & = & d^2(1 - c_\alpha)(1 - c_y c_z + 2s_x c_y). \end{array} \tag{90}$$

Moreover, using the definition of α_x

$$\alpha_x = \arctan\left(\frac{s_y s_z}{c_y + c_z}\right), \tag{15}$$

it can be shown that

$$s_x = \frac{s_y s_z}{1 + c_y c_z}, \tag{91}$$

by noting that

$$\forall \theta \in \mathbb{R}, \quad \sin(\arctan(\theta)) = \frac{\theta}{\sqrt{1 + \theta^2}}. \tag{92}$$

Therefore, by using (91) in (90), we obtain

$$\begin{array}{rcl} (y_{ee} + z_{ee})^2 & = & d^2(1 - c_\alpha)(1 - c_y c_z - \frac{2c_y s_y s_z}{1+c_y c_z}) \\ (y_{ee} - z_{ee})^2 & = & d^2(1 - c_\alpha)(1 - c_y c_z + \frac{2c_y s_y s_z}{1+c_y c_z}), \end{array} \tag{93}$$

and after simplification it yields

$$\begin{aligned} (y_{ee}+z_{ee})^2 &= \tfrac{d^2(1-c_\alpha)}{1+c_y c_z}(s_y - c_y s_z)^2 \\ (y_{ee}-z_{ee})^2 &= \tfrac{d^2(1-c_\alpha)}{1+c_y c_z}(s_y + c_y s_z)^2. \end{aligned} \tag{94}$$

which can be written, using the definition of L given in (18), as

$$\begin{aligned} (y_{ee}+z_{ee})^2 &= \tfrac{L^2}{2(1+c_y c_z)}(s_y - c_y s_z)^2 \\ (y_{ee}-z_{ee})^2 &= \tfrac{L^2}{2(1+c_y c_z)}(s_y + c_y s_z)^2. \end{aligned} \tag{95}$$

APPENDIX C

In this appendix, the derivations of the equations given in Section 3.3 of the inverse kinematics model are detailed. The calculations are relying on the following equations extracted from the Sections 3.1 and 3.2 of the article. The transformation matrix ${}^0\mathrm{T}_{ee}$ is defined as

$${}^0\mathrm{T}_{ee} = \begin{pmatrix} c_z c_y & c_z s_x s_y - s_z c_x & c_z s_y c_x + s_z s_x & x_{ee} \\ s_z c_y & s_z s_x s_y + c_z c_x & s_z s_y c_x - c_z s_x & y_{ee} \\ -s_y & s_x c_y & c_x c_y & z_{ee} \\ 0 & 0 & 0 & 1 \end{pmatrix}, \tag{5}$$

where, c_k and s_k stand respectively for $\cos(\alpha_k)$ and $\sin(\alpha_k)$, for k in $\{x, y, z\}$.

There are also the following relationships between the transformations matrices of each legs and the global one:

$${}^0\mathrm{T}_{ee} = {}^0\mathrm{T}_{A_{ee}} = {}^0\mathrm{T}_{B_{ee}} = {}^0\mathrm{T}_{C_{ee}}. \tag{8}$$

And the global transformation matrix can be simplified as

$$\begin{aligned}
T_{11} &= -g_1(q_1, q_2, \alpha) \\
T_{21} &= -s_\eta g_2(q_1, q_2, \alpha) + c_\eta g_3(q_1, q_2, \alpha) \\
T_{31} &= s_\eta g_3(q_1, q_2, \alpha) + c_\eta g_2(q_1, q_2, \alpha) \\
T_{12} &= s_\eta g_2(q_1, q_2, \alpha) - c_\eta g_3(q_1, q_2, \alpha) \\
T_{22} &= -2c_\eta s_\eta g_4(q_1, q_2, \alpha) + c_\eta^2 g_5(q_1, q_2, \alpha) + s_\eta^2 g_6(q_2, \alpha) \\
T_{32} &= c_\eta s_\eta g_7(q_1, q_2, \alpha) + (c_\eta^2 - s_\eta^2) g_4(q_1, q_2, \alpha) \\
T_{13} &= -c_\eta g_2(q_1, q_2, \alpha) - s_\eta g_3(q_1, q_2, \alpha) \\
T_{23} &= c_\eta s_\eta g_7(q_1, q_2, \alpha) + (c_\eta^2 - s_\eta^2) g_4(q_1, q_2, \alpha) \\
T_{33} &= -2c_\eta s_\eta g_4(q_1, q_2, \alpha) + s_\eta^2 g_5(q_1, q_2, \alpha) + c_\eta^2 g_6(q_2, \alpha) \\
T_{14} &= -d h_1(q_1, q_2, \alpha) \\
T_{24} &= -d(c_\eta h_2(q_1, q_2, \alpha) + s_\eta h_3(q_2, \alpha)) \\
T_{34} &= d(c_\eta h_3(q_2, \alpha) - s_\eta h_2(q_1, q_2, \alpha)),
\end{aligned} \tag{10}$$

where $(g_i)_{i\in\{1,...,7\}}$ is a family of functions defined as

$$\left\{\begin{array}{rcl} g_1(q_1,q_2,\alpha) & = & c_1^2(c_2^2 - s_2^2 c_\alpha) + s_1^2 c_\alpha + 2c_1 s_1 s_2 s_\alpha \\ g_2(q_1,q_2,\alpha) & = & c_2(s_1 s_\alpha - c_1 s_2(1+c_\alpha)) \\ g_3(q_1,q_2,\alpha) & = & (c_1^2 - s_1^2)s_2 s_\alpha + c_1 s_1(2c_\alpha - c_2^2(1+c_\alpha)) \\ g_4(q_1,q_2,\alpha) & = & c_2(c_1 s_\alpha + s_1 s_2(1+c_\alpha)) \\ g_5(q_1,q_2,\alpha) & = & c_1^2 c_\alpha + s_1^2(c_2^2 - s_2^2 c_\alpha) - 2c_1 s_1 s_2 s_\alpha \\ g_6(q_2,\alpha) & = & s_2^2 - c_2^2 c_\alpha \\ g_7(q_1,q_2,\alpha) & = & (c_2^2 - s_2^2)(1+c_\alpha) + c_1^2(s_2^2(1+c_\alpha) - (1-c_\alpha)) - 2c_1 s_1 s_2 s_\alpha, \end{array}\right. \tag{11}$$

and $(h_i)_{i\in\{1,2,3\}}$ is a family of functions defined as

$$\left\{\begin{array}{rcl} h_1(q_1,q_2,\alpha) & = & c_1 s_2 s_\alpha - s_1(1-c_\alpha) \\ h_2(q_1,q_2,\alpha) & = & c_1(1-c_\alpha) + s_1 s_2 s_\alpha \\ h_3(q_2,\alpha) & = & c_2 s_\alpha. \end{array}\right. \tag{12}$$

As already stated Section 3.3, the idea of the proof is to isolate $h_3(q_2,\alpha)$ to obtain q_2. From (5), (8) and (10), we have

$$\left\{\begin{array}{rcl} y_{ee} & = & -d(c_\eta h_2 + s_\eta h_3) \\ z_{ee} & = & d(c_\eta h_3 - s_\eta h_2) \end{array}\right. . \tag{96}$$

Therefore, we obtain

$$h_3(q_2,\alpha) = \frac{c_\eta z_{ee} - s_\eta y_{ee}}{d}. \tag{97}$$

So, by using (12), we obtain

$$c_2 = \frac{1}{ds_\alpha}(c_\eta z_{ee} - s_\eta y_{ee}). \tag{98}$$

Secondly, to obtain q_1, the idea is to extract a system in c_1 and s_1 from the last column of ${}^0\mathrm{T}_{l_{ee}}$.

From (5) and (8), we have

$$\begin{pmatrix} x_{ee} \\ y_{ee} \\ z_{ee} \end{pmatrix} = {}^0\mathrm{T}_{l_{ee}}(1:3,4). \tag{99}$$

Therefore, using (10) and (12), we obtain

$$\left\{\begin{array}{rcl} x_{ee} & = & d(1-c_\alpha)s_1 - ds_2 s_\alpha \\ y_{ee} & = & -dc_\eta(1-c_\alpha)c_1 - ds_2 s_\alpha c_\eta s_1 - dc_2 s_\alpha s_\eta \\ z_{ee} & = & -ds_\eta(1-c_\alpha)c_1 - ds_2 s_\alpha s_\eta s_1 + dc_2 s_\alpha c_\eta \end{array}\right. . \tag{100}$$

Therefore, we can obtain the following system

$$\begin{pmatrix} x_{ee} \\ -(c_\eta y_{ee} + s_\eta z_{ee}) \end{pmatrix} = \begin{pmatrix} d(1-c_\alpha) & -ds_2 s_\alpha \\ ds_2 s_\alpha & d(1-c_\alpha) \end{pmatrix} \begin{pmatrix} s_1 \\ c_1 \end{pmatrix}. \tag{101}$$

The first matrix on the right side of (101) is invertible as its determinant, denoted $\tilde{d}$, is equal to $d((1-c_\alpha)^2 + (s_2 s_\alpha)^2)$ which cannot be equal to 0 as long as $\alpha \neq 0 \quad [2\pi]$. This is the case in this system, as α represents the angular deviation of the middle linkage of the leg and cannot be null. So, by inverting this matrix, we obtain

$$\begin{pmatrix} s_1 \\ c_1 \end{pmatrix} = \frac{1}{\tilde{d}} \begin{pmatrix} 1-c_\alpha & s_2 s_\alpha \\ -s_2 s_\alpha & 1-c_\alpha \end{pmatrix} \begin{pmatrix} x_{ee} \\ -(c_\eta y_{ee} + s_\eta z_{ee}) \end{pmatrix}. \tag{102}$$

APPENDIX D

Using the complete solution of the inverse kinematics problem provided in Section 3.3, an explicit formulation of the matrix $\mathbf{A}_u$, defined in

$$\mathbf{A}_u = \frac{\partial f_{IK}}{\partial u}(\mathbf{u}) = \begin{pmatrix} \partial_{\alpha_y} f_{IK,A}(\mathbf{u}) & \partial_{\alpha_z} f_{IK,A}(\mathbf{u}) \\ \partial_{\alpha_y} f_{IK,B}(\mathbf{u}) & \partial_{\alpha_z} f_{IK,B}(\mathbf{u}) \\ \partial_{\alpha_y} f_{IK,C}(\mathbf{u}) & \partial_{\alpha_z} f_{IK,C}(\mathbf{u}), \end{pmatrix}, \tag{33}$$

can be computed as

$$\mathbf{A}_u = \begin{pmatrix} A_{11} & A_{12} \\ A_{21} & A_{22} \\ A_{31} & A_{32} \end{pmatrix}, \tag{103}$$

where all the terms are defined as

$$\begin{aligned}
A_{11} &= -f_1 \tfrac{c_A(c_y+c_z)-s_A s_y s_z+(c_A c_y - s_A s_y s_z)(1+c_y c_z)}{2s_{A2}(1+c_y c_z)^2[(1+c_\alpha)s_{A2}^2+1-c_\alpha]} - \tfrac{c_A s_y s_z + s_A(c_y+c_z)}{(1+c_y c_z)^2+(c_A c_y s_z - s_A s_y)^2} \\
A_{12} &= -f_1 c_y \tfrac{s_A(c_y+c_z)+c_A s_y s_z+s_A c_z(1+c_y c_z)}{2s_{A2}(1+c_y c_z)^2[(1+c_\alpha)s_{A2}^2+1-c_\alpha]} + \tfrac{c_y[c_A(c_y+c_z)-s_A s_y s_z]}{(1+c_y c_z)^2+(c_A c_y s_z - s_A s_y)^2} \\
A_{21} &= -f_2 \tfrac{c_B(c_y+c_z)-s_B s_y s_z+(c_B c_y - s_B s_y s_z)(1+c_y c_z)}{2s_{B2}(1+c_y c_z)^2[(1+c_\alpha)s_{B2}^2+1-c_\alpha]} - \tfrac{c_B s_y s_z + s_B(c_y+c_z)}{(1+c_y c_z)^2+(c_B c_y s_z - s_B s_y)^2} \\
A_{22} &= -f_2 c_y \tfrac{s_B(c_y+c_z)+c_B s_y s_z+s_B c_z(1+c_y c_z)}{2s_{B2}(1+c_y c_z)^2[(1+c_\alpha)s_{B2}^2+1-c_\alpha]} + \tfrac{c_y[c_B(c_y+c_z)-s_B s_y s_z]}{(1+c_y c_z)^2+(c_B c_y s_z - s_B s_y)^2} \\
A_{31} &= -f_3 \tfrac{c_C(c_y+c_z)-s_C s_y s_z+(c_C c_y - s_C s_y s_z)(1+c_y c_z)}{2s_{C2}(1+c_y c_z)^2[(1+c_\alpha)s_{C2}^2+1-c_\alpha]} - \tfrac{c_C s_y s_z + s_C(c_y+c_z)}{(1+c_y c_z)^2+(c_C c_y s_z - s_C s_y)^2} \\
A_{32} &= -f_3 c_y \tfrac{s_C(c_y+c_z)+c_C s_y s_z+s_C c_z(1+c_y c_z)}{2s_{C2}(1+c_y c_z)^2[(1+c_\alpha)s_{C2}^2+1-c_\alpha]} + \tfrac{c_y[c_C(c_y+c_z)-s_C s_y s_z]}{(1+c_y c_z)^2+(c_C c_y s_z - s_C s_y)^2}.
\end{aligned} \tag{104}$$

The terms f_1, f_2, and f_3 are defined as

$$\begin{cases} f_1 = t_{\alpha/2}(c_A s_y + s_A c_y s_z) \\ f_2 = t_{\alpha/2}(c_B s_y + s_B c_y s_z) \\ f_3 = t_{\alpha/2}(c_C s_y + s_C c_y s_z) \end{cases}, \tag{105}$$

where $t_{\alpha/2}$ stands for $\tan(\frac{\alpha}{2})$.
